# Kernel-imbedded Gaussian processes for disease classification using microarray gene expression data

**DOI:** 10.1186/1471-2105-8-67

**Published:** 2007-02-28

**Authors:** Xin Zhao, Leo Wang-Kit Cheung

**Affiliations:** 1 Department of Information and Computer Sciences, University of Hawaii, 1680 East-West Road, Honolulu, Hawaii, 96822 USA; 2 Bioinformatics Core, Stritch School of Medicine, Loyola University Medical Center, 2160 South First Avenue, Maywood, Illinois 60153 USA; 3 Department of Preventive Medicine and Epidemiology, Stritch School of Medicine, Loyola University Medical Center, 2160 South First Avenue, Maywood, Illinois 60153 USA

## Abstract

**Background:**

Designing appropriate machine learning methods for identifying genes that have a significant discriminating power for disease outcomes has become more and more important for our understanding of diseases at genomic level. Although many machine learning methods have been developed and applied to the area of microarray gene expression data analysis, the majority of them are based on linear models, which however are not necessarily appropriate for the underlying connection between the target disease and its associated explanatory genes. Linear model based methods usually also bring in false positive significant features more easily. Furthermore, linear model based algorithms often involve calculating the inverse of a matrix that is possibly singular when the number of potentially important genes is relatively large. This leads to problems of numerical instability. To overcome these limitations, a few non-linear methods have recently been introduced to the area. Many of the existing non-linear methods have a couple of critical problems, the model selection problem and the model parameter tuning problem, that remain unsolved or even untouched. In general, a unified framework that allows model parameters of both linear and non-linear models to be easily tuned is always preferred in real-world applications. Kernel-induced learning methods form a class of approaches that show promising potentials to achieve this goal.

**Results:**

A hierarchical statistical model named kernel-imbedded Gaussian process (KIGP) is developed under a unified Bayesian framework for binary disease classification problems using microarray gene expression data. In particular, based on a probit regression setting, an adaptive algorithm with a cascading structure is designed to find the appropriate kernel, to discover the potentially significant genes, and to make the optimal class prediction accordingly. A Gibbs sampler is built as the core of the algorithm to make Bayesian inferences. Simulation studies showed that, even without any knowledge of the underlying generative model, the KIGP performed very close to the theoretical Bayesian bound not only in the case with a linear Bayesian classifier but also in the case with a very non-linear Bayesian classifier. This sheds light on its broader usability to microarray data analysis problems, especially to those that linear methods work awkwardly. The KIGP was also applied to four published microarray datasets, and the results showed that the KIGP performed better than or at least as well as any of the referred state-of-the-art methods did in all of these cases.

**Conclusion:**

Mathematically built on the kernel-induced feature space concept under a Bayesian framework, the KIGP method presented in this paper provides a unified machine learning approach to explore both the linear and the possibly non-linear underlying relationship between the target features of a given binary disease classification problem and the related explanatory gene expression data. More importantly, it incorporates the model parameter tuning into the framework. The model selection problem is addressed in the form of selecting a proper kernel type. The KIGP method also gives Bayesian probabilistic predictions for disease classification. These properties and features are beneficial to most real-world applications. The algorithm is naturally robust in numerical computation. The simulation studies and the published data studies demonstrated that the proposed KIGP performs satisfactorily and consistently.

## Background

DNA microarray technology provides researchers a high-throughput means to measure expression levels for thousands of genes in an experiment. Careful analyses of microarray gene expression data can help better understand human health and disease and have very important implications in basic sciences as well as pharmaceutical and clinical research. Some existing methodologies for microarray gene expression data analysis, such as introduced in [1-3] and [4], have demonstrated their usefulness for a variety of class discovery or class prediction problems in biomedical applications. In a microarray study, we typically face a problem of analyzing thousands of genes from a relatively small number of available samples. This nature gives rise to a very high likelihood of finding lots of "false positives" with conventional statistical methods. Therefore, properly selecting the group of genes that are significantly related to a target disease has created one of the key challenges in microarray data analysis.

Gene selection problem basically can be viewed as a variable selection problem associated with linear regression models. An incomplete list of those classical variable selection methods/criteria includes the ratio of error sum of squares for the model with p variables to the error mean square of the full model and adjusted with a penalty for the number of variables or the *C*_*p *_Criterion [5], the Akaike Information Criterion or AIC [6], and the Bayesian Information Criterion or BIC [7]. George and Foster [8] later suggested that these criteria corresponded to a hierarchical Bayesian variable selection procedure under a particular class of priors. Following the similar setting with a slightly different prior specification, Yuan and Lin [9] provided another approach to solve this problem and they showed that their algorithm was significantly faster and could be potentially used even when the predictor dimension is larger than the training sample size. Although both of these algorithms have been shown to favorably enhance the selection performance comparing to the classical methods such as *C*_*p*_, AIC or BIC, they share a common disadvantage. That is, even after the hyperparameters are estimated, the variable selection criteria need to be evaluated on each candidate variable for optimality. Usually, the number of candidate models grows in an exponential rate with the increase of the number of variables, whereas the typical number of the investigated genes in a microarray data analysis problem is in thousands. This motivates the development of the class of the Markov Chain Monte Carlo (MCMC) algorithms under a Bayesian framework to attack the problem. One of the most widely used MCMC algorithms is the Gibbs sampler. For the microarray analysis problem, Lee et al. [10] suggested a Bayesian model based on a linear probit regression setting and proposed a Gibbs Sampler to solve it. An extension to this method based on a multinomial probit regression setting has also been proposed [11]. Similarly, Zhou et al. ([12,13]) developed another Bayesian approach built upon a linear logistic regression model to the gene selection problem.

The linear model based methods mentioned above have been shown with various levels of effectiveness in finding the set of significant genes in a wide range of real microarray experiments. However, they all share some common limitations: the first also the most important one is that, a linear model is not necessarily always a good approximation for the underlying physical model; second, linear model based methods are more likely to bring in false positives; third, the computations of these linear model based algorithms usually involve calculating the inverse of a matrix that is possibly singular when the number of potentially important genes is relatively large. To overcome these disadvantages, Zhou et al. [14] introduced a non-linear term into the basic linear probit regression model and applied a bootstrapping procedure to enlarge the sample size. A technique called sequential Monte Carlo was adopted in the numerical Bayesian computation in their work. Some other models were also developed for tumor classification problems with gene expression profiling. For instance, based on the simple nearest centroid classifier and via a shrinking strategy, Tibshirani et al. [15] offered the so-called "nearest shrunken centroids" (also known as "Prediction Analysis for Microarrays" or PAM) algorithm. By combining two ensemble schemes, i.e. bagging and boosting, Dettling [16] introduced the method "BagBoosting" as an enhanced version of the regular boosting algorithm. Both of these methods were shown being very effective when applied to a few published datasets.

The kernel-induced machine learning is one of the most promising approaches for exploring the potential non-linearity for a given classification or regression problem through the feature space concept. For example, kernel-induced support vector machines (SVMs) have been successfully applied to a number of learning tasks and are generally accepted as one of the state-of-the-art learning methods. Theoretically, Lin et al. ([17,18]) proved that a SVM with an appropriately chosen kernel and model parameters can approach the Bayesian bound of a given problem when the training sample size is large enough. For the gene-selection problem, Guyon et al. [19] proposed the method "Recursive Feature Elimination" (RFE) to rank the genes with respect to a provided SVM, thus the SVM can be utilized for microarray data analysis. RFE was shown to be very effective with a linear kernel. However, when the number of genes is large (in hundreds), RFE doesn't function well with a non-linear kernel. This limits the applications of SVMs to the analysis of microarray data. Zhu and Hastie ([20,21]) later proposed a framework called kernel logistic regression and suggested a method called "Import Vector Machine" to solve it. However, they also chose the RFE as the strategy to select the significant genes.

As Bayesian probability theory can help construct a unified framework for modeling data and facilitate tuning of the involved parameter and/or hyperparameter, developing a proper Bayesian probabilistic model is usually beneficial for a machine learning method. MacKay [22] introduced a probabilistic evidence framework as a Bayesian learning paradigm for neural networks. With the close relationship between neural network methods and kernel-induced learning methods, Kwok [23] and Gestel et al. [24] developed a Bayesian framework for SVMs and least square support vector machines (LSSVMs) respectively, with guidance of the principle of the evidence framework. Neal [25] also showed that, as the number of hidden units increases in a Bayesian neural network, the prior over the network output converges to a Gaussian process (GP) if independent Gaussian distributions are used as the priors for network weights and bias. LSSVMs conceptually are close to SVMs, except that they use equality constraints instead of inequality constraints and they use a squared error penalty function. Getting solution of an LSSVM therefore only involves solving a set of linear equations, which though loses the sparseness featured in an SVM, it makes an LSSVM much easier for an on-line implementation. If we consider the characteristic similarity between the mapping from input nodes/data to hidden units in a neural network and the mapping from input data to a feature space conceptually embedded in an LSSVM, it's not surprising that under the Gaussian noise assumption, the mean of the posterior prediction made by a GP coincides with the optimum decision function made by an LSSVM, whereas a GP offers a more approachable probabilistic model. This fact motivated us to develop a new Bayesian learning method named kernel-imbedded Gaussian process (KIGP) for microarray gene expression data analysis based on the Gaussian process theory.

**Figure 1:**
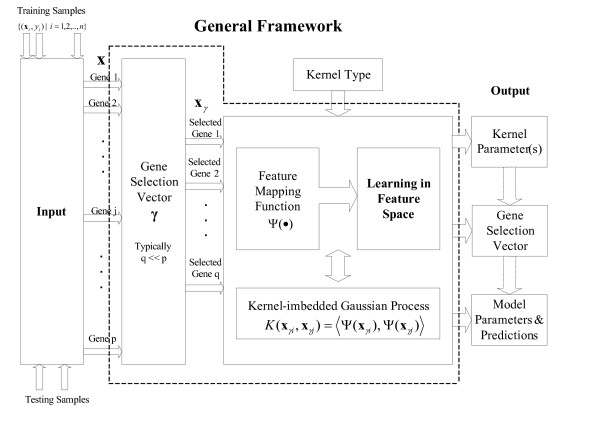
Schematic plot for the general framework of the proposed KIGP method.

The general framework of the KIGP method is sketched in Fig. 1, where the box bounded by the dotted lines represents the proposed learning component of the method. Conceptually, via a gene-selection procedure, a small group of the gene data is selected. Through a feature mapping function Ψ(·), the selected gene data are mapped into a feature space where the optimal classification procedure is processed. With the theory of kernel-induced feature space [26], we do not really do the feature mapping computationally. Instead, we train the data via a kernel-imbedded Gaussian Process by using a kernel function. In the output end, there are basically three consecutive phases, the "kernel parameter fitting phase", the "gene selection phase", and the "prediction phase". Given a kernel type, the KIGP algorithm finds the fitted kernel parameter(s) in the "kernel parameter fitting phase". After fixing the kernel parameter(s) at the fitted value(s), it continues with the "gene selection phase" and yields a group of significant genes under some given confidence level. Based on the fitted kernel parameter(s) and the selected significant gene data, the algorithm makes a probabilistic prediction for each testing sample in the "prediction phase". The details of the algorithm are discussed in the "Methods" section.

The rest of this paper is organized as follows: we show the results from applying the proposed KIGP method to simulated datasets as well as real published microarray datasets in the "Results" section. The conclusions and the further research discussions are summarized in the "Discussions and Conclusions" section. In the "Methods" section, we provide the mathematical content of the methodology followed by a detailed description of the algorithm.

## Results

Some terms and acronyms defined in the "Methods" section are used in this section. They include "gene-selection vector (*γ*)", "linear kernel (LK)", "polynomial kernel (PK)", "Gaussian kernel (GK)", "Normalized Log-Frequency (NLF)", "false discovery rate (fdr)", "kernel parameter fitting phase", "gene selection phase", "prediction phase", "misclassification rate (MR)", "average predictive probability (APP)", "leave-one-out cross-validation (LOOCV)" and "3-fold cross-validation (3-fold CV)". One can refer to the "Methods" section for the details.

### Simulation studies

#### Example 1

This example was designed to illustrate all the key concepts, elements and procedures of the KIGP framework introduced in the "Methods" section. It consists of two cases. In the first case, the Bayesian classifier of the underlying generative model is linear; while in the second case, the Bayesian classifier takes a very non-linear form. We set the number of the significant/explanatory genes as two, so we can better graphically display the Bayesian classifier and the relative performance of the KIGP method. In both of these cases, the number of training samples is twenty. Ten training samples were generated from the class "1" and the other ten samples were generated from the class "-1". The number of testing samples is 5000. For each sample, the number of investigated genes is 200; the indices of the two underlying explanatory genes were preset as [23,57]. For each case, we independently generated 10 sets of training samples from the generative model and ran the simulation on each of them.

#### (a) Case with a Linear Bayesian Classifier

In this linear case, the two preset significant genes were generated from the bivariate Gaussian distribution N([11],[1−1−12])
 MathType@MTEF@5@5@+=feaafiart1ev1aaatCvAUfKttLearuWrP9MDH5MBPbIqV92AaeXatLxBI9gBaebbnrfifHhDYfgasaacH8akY=wiFfYdH8Gipec8Eeeu0xXdbba9frFj0=OqFfea0dXdd9vqai=hGuQ8kuc9pgc9s8qqaq=dirpe0xb9q8qiLsFr0=vr0=vr0dc8meaabaqaciaacaGaaeqabaqabeGadaaakeaacqWGobGtcqGGOaakdaWadaqaauaabeqaceaaaeaacqaIXaqmaeaacqaIXaqmaaaacaGLBbGaayzxaaGaeiilaWYaamWaaeaafaqabeGacaaabaGaeGymaedabaGaeyOeI0IaeGymaedabaGaeyOeI0IaeGymaedabaGaeGOmaidaaaGaay5waiaaw2faaiabcMcaPaaa@3BE0@ for the class "1" and from the bivariate Gaussian distribution N([−1−1],[1−1−12])
 MathType@MTEF@5@5@+=feaafiart1ev1aaatCvAUfKttLearuWrP9MDH5MBPbIqV92AaeXatLxBI9gBaebbnrfifHhDYfgasaacH8akY=wiFfYdH8Gipec8Eeeu0xXdbba9frFj0=OqFfea0dXdd9vqai=hGuQ8kuc9pgc9s8qqaq=dirpe0xb9q8qiLsFr0=vr0=vr0dc8meaabaqaciaacaGaaeqabaqabeGadaaakeaacqWGobGtcqGGOaakdaWadaqaauaabeqaceaaaeaacqGHsislcqaIXaqmaeaacqGHsislcqaIXaqmaaaacaGLBbGaayzxaaGaeiilaWYaamWaaeaafaqabeGacaaabaGaeGymaedabaGaeyOeI0IaeGymaedabaGaeyOeI0IaeGymaedabaGaeGOmaidaaaGaay5waiaaw2faaiabcMcaPaaa@3DBA@ for the class "-1". For those insignificant genes, each of them was independently generated from the standard normal distribution *N*(0,1). The probabilities for the class "1" and the class "-1" were equal. With this generative model, the Bayesian classifier for the two classes is a mathematical linear combination of the two prescribed significant genes.

The KIGP method with an PK, or with an GK, or with an LK was applied to each of the 10 training sets respectively. The prior probability for *γ*_*j *_= 1 for all *j *in the Gibbs sampling simulations was set at 0.01. For all the Gibbs sampling simulations in this example, we ran 5000 iterations in both the "kernel parameter fitting phase" and the "gene selection phase" and treated the first 1000 iterations as the burn-in period. In the "prediction phase", we ran 2000 iterations and treated the first 500 iterations as the burn-in period. The threshold for "fdr" in the "gene selection phase" was set at 0.05.

For all of the 10 simulated training sets, when an PK was the kernel type for the KIGP method, the algorithm chose the PK(1) after the "kernel parameter fitting phase" and found both the prescribed significant genes at the end of the "gene selection phase" (i.e. with no "false negative"). However, KIGP with an PK(1) resulted with one "false positive" gene in 2 of the 10 sets. In the prediction phase, the average testing MR for the 8 sets correctly found the 2 preset significant genes with no "false positive" was 0.018. It was very close to the Bayesian bound (i.e. 0.013). However, the average testing MR for the 2 sets with one "false positive" was significantly worse. It was only 0.107. The average testing MR for all 10 sets was 0.036.

The results of the simulation studies with an LK were very similar to that of the simulations with the PK(1). In all the simulations, the KIGP found the 2 preset significant genes (i.e. with no false "negative"), but in 2 of the 10 sets, the algorithm resulted with one "false positive" as well. This result was exactly same as that from the simulations with the PK(1). The average testing MR for the 10 sets with an LK was 0.037, almost the same as to that with an PK.

For the results of the stimulation studies with an GK, the algorithm perfectly found the only 2 prescribed significant genes in 6 of the 10 sets (i.e. no false "negative" and no false "positive"). In other 3 sets, the KIGP identified the 2 prescribed significant genes as well as one "false positive". In one other set, the KIGP resulted only one of the two prescribed genes (i.e. with one "false negative") and one "false positive". The mean and the standard deviation for the fitted width of an GK for these 10 simulations were 1.95 and 0.31 respectively. The average testing MR for the 10 simulations with an GK was only 0.104. Based on the testing MR measure, we should use the KIGP with either a polynomial kernel or a linear kernel to make any further analysis for this problem.

**Figure 2:**
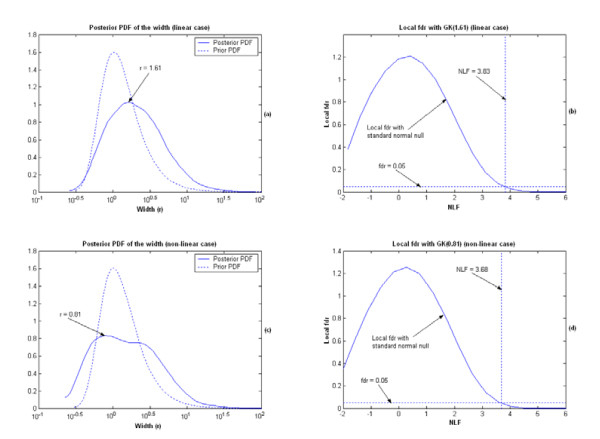
The results from applying the KIGP with an GK to one of the training sets of the simulated example 1, where (a) and (b) are for the linear case; (c) and (d) are for the non-liner case. (a) The estimated marginal posterior PDF of the width parameter of the GK (solid line) versus its prior PDF (dotted line). The mode of the posterior PDF is at around 1.61. (b) The local fdr with the GK(1.61) (with the standard normal as the density of NLF under null hypothesis); the horizontal dotted line represents the threshold of the fdr (0.05); the vertical dotted line shows the resulted cutoff value for NLF (3.83). (c) The estimated marginal posterior PDF of the width parameter of the GK (solid line) versus its prior PDF (dotted line). The mode of the posterior PDF is at around 0.81. (d) The local fdr with the GK(0.81) (with standard normal as the density of NLF under null hypothesis); the horizontal dotted line represents the threshold of the fdr (0.05); the vertical dotted line shows the resulted cutoff value for NLF (3.68).

**Figure 3:**
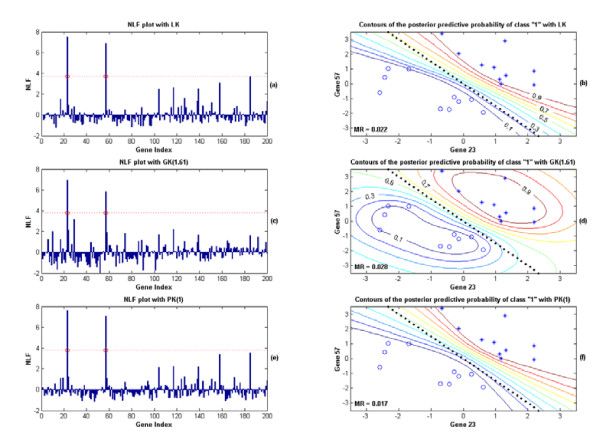
The results from applying the KIGP to one of the training sets of the linear case in the simulated example 1, where (a) and (b) are for the simulation with an LK; (c) and (d) are for the one with an GK; (e) and (f) for the one with an PK. (a) The NLF plot of each gene for the simulation with an LK; with the cutoff value for NLF (dotted line), two genes were found significant (the circles mark the preset significant genes). (b) The contours of the posterior predictive probability of the class "1" for the simulation with an LK, where X-axis is for the value of the gene 23 and Y-axis represents the value of the gene 57; the numbers associated with contours are the probabilities; the asterisks denote the training samples from the class "1"; the circles demonstrate the training samples from the class "-1"; the dotted line shows the Bayesian classifier. For this set of training samples, the testing MR is 0.022 (the Bayesian bound for MR is 0.013). (c) Same as (a) except it is for the simulation with an GK. (d) Same as (b) except it is for the simulation with an GK. The testing MR is 0.028. (e) Same as (a) except it is for the simulation with an PK. (f) Same as (b) except it is for the simulation with an PK. The testing MR is 0.017.

As an illustration, we specifically display the results from applying the KIGP to one of the training sets, in which both an PK and an GK worked very well. For the simulation with the GK, the posterior probability density function (PDF) of the width parameter "r" is plotted in Fig. 2a, in which its mode was found at around 1.61. After the "kernel parameter fitting phase", the kernel was fixed as the GK(1.61). With the posterior samples obtained in the "gene selection phase", the NLF for each gene was calculated (Fig. 3c). Following the procedure described in the "Gene selection phase" subsection, the local fdr with respect to the NLF value was estimated (Fig. 2b). With the threshold for fdr set at 0.05, the cutoff value for NLF was 3.83 and we found that only the two prescribed genes (indices: 23, 57) were found significant. The contours of the posterior predictive probabilities for the class "1" are plotted in Fig. 3d, where the X-axis is the value of the gene 23 and the Y-axis represents the value of the gene 57. In Fig. 3d, the numbers associated with the contour curves are probabilities; the asterisks denote the positive training samples and the circles present the negative training samples; the dotted line shows the Bayesian classifier. The MR of the independent testing set for this simulation was 0.028. For the simulation with an PK, after the "kernel parameter fitting phase", the estimated posterior probability masses for the discrete degree parameter "d" were Prob(d = 1) = 0.797 and Prob(d = 2) = 0.203 respectively. With the highest estimated posterior mass at d = 1, we accordingly fixed the kernel as the PK(1). With the same gene-selection procedure described in the simulation with the GK, the two prescribed genes again were found as the only two significant genes (Fig. 3e). The contour plot of the posterior predictive probability for the class "1" is drawn in Fig. 3f. The testing MR was 0.017 for this simulation. The performance of the KIGP with the PK(1) was very similar to that of the KIGP with an LK (Fig. 3a and 3b). Both of them behaved like the linear Bayesian classifier. As a benchmark comparison, we further applied a regular SVM/RFE (SVM with RFE [19] as the gene selection strategy) to each of the 10 simulated training sets. In fact, rather than using a cross-validation procedure, there is no effective way for a SVM/RFE to set the model parameter (such as the box constraint) and to select the number of significant genes. Technically, it is also important to mention that the SVM/RFE is not proper for microarray data analysis with a kernel type having variable parameter(s) such as a Gaussian kernel. Nevertheless, for this linear example, we applied a SVM/RFE with an LK to the datasets and preset the box constraint as 1. The obtained results were similar to those of the KIGP with an LK case. In 8 out of the 10 sets, the gene 23 and the gene 57 were ranked as the top 2 genes in the significance gene list. However, in the remaining 2 of the 10 sets, the gene 23 was ranked as the top significant gene but the gene 57 was ranked in the 3^rd ^place and in the 5^th ^place respectively. For the prediction with RFE, we used the top genes including the gene 23 and the gene 57. The resulted average testing MR for all 10 sets was 0.058. Even in this linear case, the KIGP with an LK or the PK(1) outperformed the SVM/RFE with an LK in an automatic fashion. More importantly, the SVM/RFE only made a binary prediction of the class for each testing sample, while the KIGP gave a probabilistic prediction on the certainty of the decision. Furthermore, the proposed KIGP framework offered the posterior distribution for each model parameter as well as a universal significance measure (NLF) for each investigated gene at the end.

In the majority of the simulations, the KIGPs found the two preset significant genes in this linear case. They all performed very close to the Bayesian bound when the two preset genes were perfectly found. Since the KIGP with the PK(1) gave the best average testing MR, we should use it for any further analysis.

#### (b) Case with a Non-linear Bayesian Classifier

**Figure 4:**
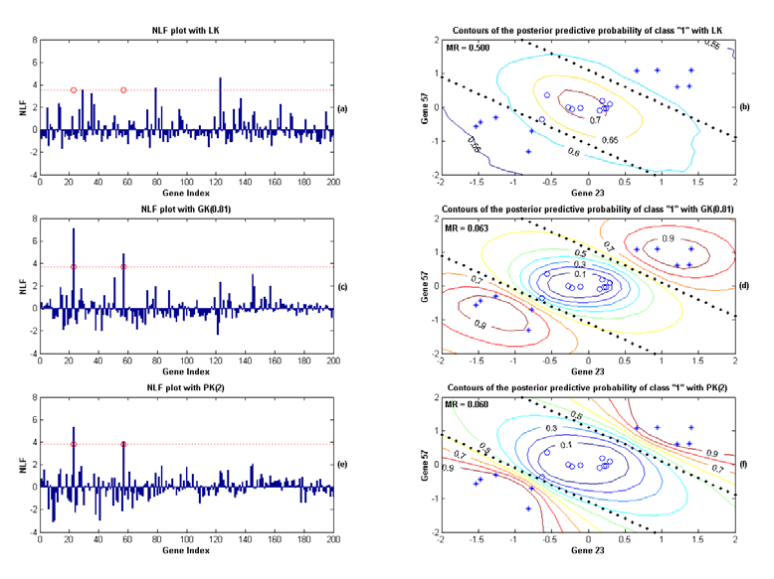
The results from applying the KIGP to one of the training sets for the non-linear case in the simulated example 1, where (a) and (b) are for the simulation with an LK; (c) and (d) are for the simulation with an GK; (e) and (f) for the simulation with an PK. All the legends are same as those in Fig. 3. (a) The NLF plot of each gene for the simulation with an LK; with the cutoff value for NLF (dotted line), none of the true preset significant genes was found (2 false negatives). Three false positive genes were misclassified as significant. (b) The contours of the posterior predictive probability of the class "1" for the simulation with an LK (given the two true preset significant genes). For this set of training samples, the testing MR is 0.5 (the Bayesian bound is 0.055). (c) Same as (a) except it is for the simulation with an GK. (d) Same as (b) except it is for the simulation with an GK. The testing MR is 0.063. (e) Same as (a) except it is for the simulation with an PK. (f) Same as (b) except it is for the simulation with an PK. The testing MR is 0.060.

In this non-linear case, the two preset significant genes were generated from a mixture Gaussian distribution with equal probability on *N*(**1**_2_, **I**_2 _*0.16) and *N*(-**1**_2_, **I**_2 _*0.16) for the class "1" and from an independent normal distribution *N*(0,0.16) for the class "-1". **1**_2 _and **I**_2 _denote the one-vector and the identity matrix respectively (defined in (7) of the "Methods" section). For those insignificant genes, each of them was independently drawn from the standard normal distribution *N*(0,1). The probabilities for the two classes were equal. The Bayesian classifier given the two significant genes looks like two parallel lines (Fig. 4) and the Bayesian bound for the MR is 0.055. We applied both the linear probit regression method proposed by Lee et al. [10] and an KIGP with an LK (such as in Fig. 4a) to the 10 training sets. Unsurprisingly, both of them failed badly in terms of finding the correct significant genes and making optimal class predictions for this non-linear case.

For the 10 simulated training sets, when an PK was the kernel type for the KIGP method, the algorithm chose the PK(1) for 5 sets and the PK(2) for the other 5 sets after the "kernel parameter fitting phase". Only in 2 of the 5 sets, the KIGP with the PK(2) perfectly found the two prescribed genes as the only significant genes. The average testing MR for these 10 sets was horrendous. However, for those two sets correctly found the two preset significant genes, the testing MRs were both fairly close to the Bayesian bound.

The results of the simulations with an GK were much better. For all of the 10 sets, the KIGP successfully found the 2 preset significant genes (i.e. with no "false negative"). The KIGP also resulted with one "false positive" for 2 sets as well. The mean and the standard deviation of the fitted width of an GK for these 10 sets were 0.71 and 0.08 respectively. In the "prediction phase", the average testing MR was 0.065 for the 8 sets correctly found the 2 preset significant genes. It was very close to the Bayesian bound (i.e. 0.055). The average testing MR was 0.171 for the 2 sets with one "false positive". The average testing MR for all 10 sets was 0.086.

As an illustration, we depict the results from applying the KIGP to one of the training sets, in which both an PK and an GK worked well. The procedure and all settings of the simulations and the legends of the figures were same as described in the linear case. We first applied an KIGP with an GK to the training set. The mode of the posterior PDF of the width parameter was found at around 0.81 after the "kernel parameter fitting phase" (Fig. 2c). With the GK(0.81), the cutoff value of 3.68 for NLF was obtained at the end of the "gene selection phase". Based on the NLF statistic, the two prescribed genes were successfully retrieved (Fig. 4c) and the KIGP performed well with MR = 0.063 (Fig. 4d). It was very close to the Bayesian bound. For the simulation with an PK, the posterior probability masses of the degree parameter were Prob(d = 1) = 0.229 and Prob(d = 2) = 0.771 respectively. The NLF plot for each gene and the relative cutoff line for the NLF are both displayed in Fig. 4e. The two prescribed genes were discovered. The performance of the KIGP with the PK(2) was very well with MR = 0.060 (Fig. 4f). It was very close to the Bayesian bound too.

We tried to apply the regular SVM with RFE to this example as we did in the linear case, but SVM/RFE failed to work with an LK, nor an GK (with any width), nor an PK. The key problem might be due to the large dimension (i.e. 200) of this example. Comparing the KIGP method to the SVM/RFE in this non-linear case, besides those beneficial properties of the KIGP that we already observed in the linear case, the KIGP method particularly shows its better adaptability for non-linear problems. In summary, owing to the non-linear setting of this case, all linear methods were not applicable. The regular SVM/RFE approach also did not work. On the contrary, in terms of the testing MR measure, the KIGP with an GK provided a performance very close to the Bayesian bound. Comparatively, the KIGP with an PK seems to be less robust and consistent than the KIGP with an GK for a non-linear problem in general.

As a side note, it's worth pointing out that the posterior PDF of the width parameter seems to disclose some special nature of a dataset for a classification problem when one applies the KIGP with an GK. For instance, we observed that if the underlying Bayesian classifier can be well approximated by a linear function, the mode (peak) of the PDF of the width parameter significantly moves to the right side of the value 1 (Fig. 2a); whereas if the Bayesian classifier is very non-linear, it moves to the left side of the value 1 (Fig. 2c).

#### Example 2

We further designed this example to demonstrate the effectiveness of the proposed KIGP method when the number of investigated genes is large, especially for a problem with a very non-linear Bayesian classifier. A total of 1000 genes in a simulated microarray experiment and 10 of them were preset as the significant genes with indices [64,237,243,449,512,573,783,818,890,961]. These 10 significant genes were generated from the mixture Gaussian distribution with equal probability on *N*(**1**_10_, **I**_10 _*0.1) and *N*(-**1**_10_, **I**_10 _*0.1) for the class "1" and from the Gaussian distribution *N*(**0**_10_, **I**_10 _*0.1) for the class "-1", where **0**_10 _denotes a vector with 10 "0" elements. The probabilities for the two classes were equal. The rest of other insignificant genes were independently generated from the standard normal distribution *N*(0,1). Similar to the first example, the number of training samples is 20, 10 of which were generated from the class "1" and the other 10 samples were generated from the class "-1"; the number of testing samples is 5000; we independently generated 10 sets of training samples from the model and ran the simulation on each of them.

The procedure for this example is same as in the non-linear case of the first example. The prior probability for *γ*_*j *_= 1 was set at 0.01. For both the "kernel parameter fitting phase" and the "gene selection phase", we ran 20000 iterations and treated the first 10000 as the burn-in period, and for the "prediction phase", we ran 5000 iterations and treated the first 1000 as the burn-in period.

For the 10 simulated training sets, when an PK was the kernel type for the KIGP method, the algorithm chose the PK(2) in 7 out of 10 sets. Only in 2 of these 7 sets with the PK(2), the algorithm found all 10 significant genes. However, for the 10 sets with an GK, the 10 prescribed genes were all found in each of the 10 sets. There was one "false positive" being brought into the significant group in one set. There was almost no error for the testing samples and extremely close to the Bayesian bound.

**Figure 5:**
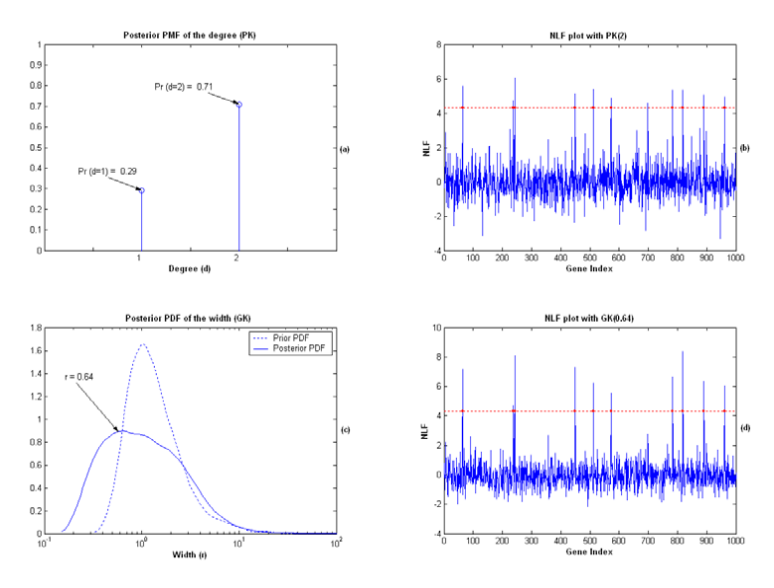
The results from applying the KIGP to one of the training sets of the simulated example 2, where (a) and (b) are for the simulation with an PK; (c) and (d) are for the simulation with an GK. (a) The estimated marginal posterior PMF of the degree parameter d. (b) The NLF plot of each gene for the simulation with the PK(2); the dots mark the prescribed significant genes. For this training set, all 10 preset significant genes and 1 false positive gene were found. (c) The estimated marginal posterior PDF of the width parameter r (solid line) versus its prior PDF (dotted line). The mode of the posterior PDF is at around 0.64. (d) The NLF plot for each gene for the simulation with the GK(0.64). The legends are same as those in (b). For this training set, all 10 preset significant genes were found with no false positive result.

In Fig. 5, we show the simulation results from applying the KIGP method to one of the training sets. Fig. 5a and 5b are for the simulation with an PK, whereas Fig. 5c and 5d are for the simulation with an GK. Based on Fig. 5a, the PK(2) was chosen after the "kernel parameter fitting phase". After the "gene selection phase", with the yielded cutoff line for the NLF, the KIGP found all 10 prescribed significant genes and one "false positive" (Fig. 5b). The MR of the testing set was 0.991. In the simulation with an GK, the mode of the posterior PDF for the width was found at around 0.64 (Fig. 5c). With the GK(0.64), after the "gene selection phase", all 10 prescribed genes were correctly found with no "false positive". With the found significant genes, we did not find any testing error in the "prediction phase". Based on the testing MR, we should choose the GK for further analysis. This example not only illustrates the usefulness of the proposed algorithm for problems with very large number of investigated genes, but also reinforces all the arguments we have made for the Bayesian KIGP framework in the last example.

### Real data studies

Following the similar procedure executed in the simulated studies, the KIGP was applied to four published microarray gene expression datasets. A brief summary of these datasets is provided in Table 1 and the experimental details are extracted and described below.

**Table 1:** Summary of the real dataset studied in this paper

**Dataset**	**Publication**	**p**	**n**	**M**	**Response**
Leukemia	Golub et al. (1999) [1]	7129	38	34	ALL/AML
SRBCT	Khan et al. (2001) [27]	2308	35	12	EWS/NB
Breast Cancer	Hedenfalk et al. (2001) [28]	3226	22	0	BRCA1/BRCA2 or sporadic
Colon	Alon et al. (1999) [30]	2000	62	0	Tumor/Normal tissue

#### Acute leukemia data

The leukemia dataset was originally published by Golub et al. [1], in which the bone marrow or peripheral blood samples were taken from 72 patients with either acute myeloid leukemia (AML) or acute lymphoblastic leukemia (ALL). The data was divided into two independent sets: a training set and a testing set. The training set consists of 38 samples, of which 27 are ALL and 11 are AML. The testing set consists of 34 samples, of which 20 are ALL and 14 are AML. This dataset contains expression levels for 7129 human genes produced by Affymetrix high-density oligonucleotide micorarrays. The scores in the dataset represent the intensity of gene expression after being rescaled. By using a weighted voting scheme, Golub et al. made predictions for all the 34 testing samples and 5 of them were reported being misclassified.

The KIGP with an GK, an PK, and an LK was applied to the training dataset (including all investigated genes) respectively. The prior parameter *π*_*j *_for all *j *was uniformly set at 0.001. In both the "kernel parameter fitting phase" and the "gene selection phase", we ran 30000 iterations and treated the first 15000 iterations as the burn-in period; and in the "prediction phase", we ran 5000 iterations and treated the first 1000 iterations as the burn-in period.

For the simulation with an PK, the resulted posterior probability masses of the degree parameter d are Prob(d = 1) = 0.985 and Prob(d = 2) = 0.015. With the PK(1), 20 genes were identified as "significant" at 0.05 significance level (Table 3). Using the PK(1) and the found significant genes, we made predictions for the 34 testing samples. We then ran a leave-one-out cross-validation (LOOCV) for the 38 training samples. This "loose" LOOCV procedure was however only involved in the "prediction phase". Since the fitted kernel parameter and the significant genes chosen from the first two phases had already contained the most information of the whole training dataset, it was not a proper validation measure for kernel type competition. More properly, we further did a rigorous 3-fold cross-validation (3-fold CV) that included all 3 phases of the proposed algorithm (the details are described in the "Kernel type competition" subsection). This whole procedure was then repeated for the simulation with an GK and with an LK respectively. All the results are summarized in Table 2.

**Table 2:** Summary of the results from applying the proposed KIGP to the leukemia dataset.

**Performance Measure**	**Test**			**CV (3-fold)**			**LOOCV (fixed genes)**		
	
	**PK**	**GK**	**LK**	**PK**	**GK**	**LK**	**PK**	**GK**	**LK**
**ERR #**	2/34	1/34	1/34	2/38	1/38	1/38	0/38	0/38	0/38
**APP**	0.858	0.835	0.923	0.844	0.819	0.875	0.995	0.928	0.993

The columns labeled by "Test" are for the independent tests.

The columns labeled by "CV (3-fold)" are for the rigorous 3-fold CVs (each CV involves all three phases of an KIGP).

The columns labeled by "LOOCV (fixed genes)" are for the loose LOOCVs (each CV only involves the "prediction phase" of an KIGP).

**Table 3:** Summary of the genes found by applying the KIGP with the LK to the leukemia dataset

**Index**	**NLF**	**Accession #**	**Gene Description**
4847	11.47	X95735	Zyxin
3320	10.36	U50136	Leukotriene C4 synthase (LTC4S) gene
2020	9.79	M55150	FAH Fumarylacetoacetate
5039	9.63	Y12670	LEPR Leptin receptor
1834	9.22	M23197	CD33 CD33 antigen (differentiation antigen)
4499*	6.79	X70297	CHRNA7 Cholinergic receptor, nicotinic, alpha polypeptide 7
1745	6.46	M16038	LYN V-yes-1 Yamaguchi sarcoma viral related oncogene homolog
3847	5.32	U82759	GB DEF = Homeodomain protein HoxA9 mRNA
4196	5.21	X17042	PRG1 Proteoglycan 1, secretory granule
1779*	5.08	M19507	MPO Myeloperoxidase
6539	4.98	X85116	Epb72 gene exon 1
6376	4.80	M83652	PFC Properdin P factor, complement
3258	4.73	U46751	Phosphotyrosine independent ligand p62 for the Lck SH2 domain mRNA
2111	4.64	M62762	ATP6C Vacuolar H+ ATPase proton channel subunit
1882	4.64	M27891	CST3 Cystatin C (amyloid angiopathy and cerebral hemorrhage)
1829*	4.59	M22960	PPGB Protective protein for beta-galactosidase (galactosialidosis)
1249	4.49	L08246	INDUCED MYELOID LEUKEMIA CELL DIFFERENTIATION PROTEIN MCL1
2121	4.41	M63138	CTSD Cathepsin D (lysosomal aspartyl protease)
2288	4.28	M84526	DF D component of complement (adipsin)
1924*	4.28	M31158	PRKAR2B Protein kinase, cAMP-dependent, regulatory, type II, beta

*: Index of the Genes not reported in [1].

In Table 2, the KIGP with an LK gave the best testing performance: only 1 error was found. We found that many publications (e.g. [10,12] and [21]) reported the same testing error for this dataset as well. Only Zhou et al. [14] reported 0 testing error. However, based on the results of [14], the testing APP was only 0.83, which is much worse than that of the KIGP with an LK (i.e. the testing APP = 0.923). We suspect that this misclassified testing sample by KIGP/LK may be phenotyped incorrectly.

**Figure 6:**
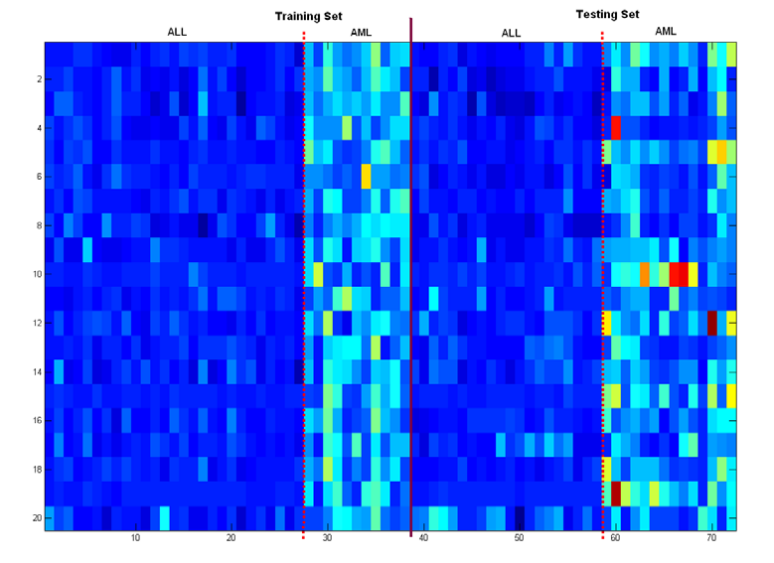
The heat map of the gene expression levels of the 20 found significant genes for the acute leukemia dataset. The panel on the left (to the solid line) represents the training samples and that on the right shows the testing samples. The two dotted lines are used to separate the two classes (ALL and AML).

**Figure 8:**
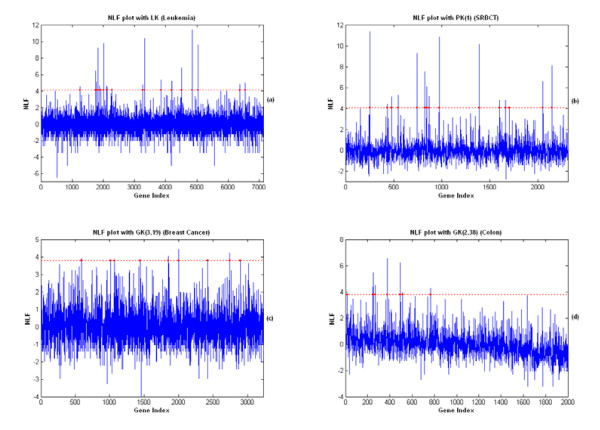
The NLF plots for all 4 real data studies with found kernels. The legends are same for all four plots. (a) The NLF plot of each gene with the LK for the leukemia dataset and the dots mark the 20 found significant genes, the details of which are listed in Table 3. (b) The NLF plot of each gene with the PK(1) for the SRBCT dataset and the details of the 15 found significant genes are listed in Table 5. (c) The NLF plot of each gene with the GK(3.19) for the breast cancer dataset and the details of the 9 found significant genes are listed in Table 7. (d) The NLF plot of each gene with the GK(2.38) for the colon dataset and the details of the 8 found significant genes are listed in Table 9.

**Figure 9:**
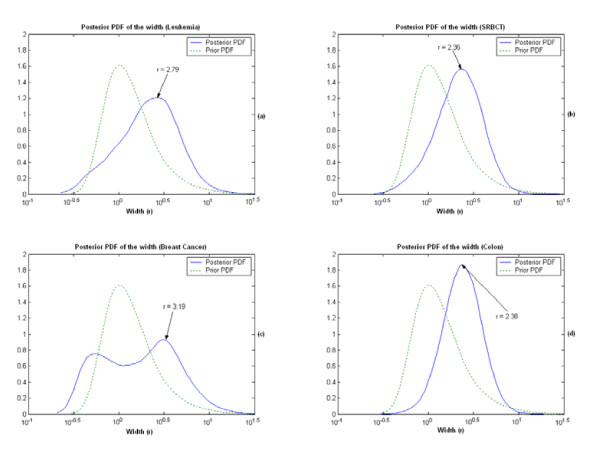
The estimated marginal posterior PDF of the width parameter of an GK for each real data study case (dotted lines present the prior PDF). (a) For the leukemia dataset, the mode of the posterior PDF is at around 2.79. (b) For the SRBCT dataset, the mode of the posterior PDF is at around 2.36. (c) For the breast cancer dataset, the mode of the posterior PDF is at around 3.19. (We also noticed that there was a local peak on the left, which was at around 0.56). (d) For the colon dataset, the mode of the posterior PDF is at around 2.38.

The significant genes found by the KIGP with an LK are reported in Table 3 and the NLF plot is plotted in Fig. 8a. In Table 3, the genes with asterisks (gene indices 4499, 1799, 1829 and 1924) are those not reported by the original paper [1]. The heat map of the found significant genes for all the samples (Fig. 6) exhibits a very good consistency between the training set and the testing set (including the genes with asterisks). We realize that the posterior PDF of the width parameter of an GK can disclose some special nature of the feature space for a given dataset and problem. Fig. 9a illustrates the dominant linearity of this case. Another issue that needs to be addressed is that, if the number of the available samples is small (often true for a typical microarray application), the measure of "the number of testing errors" may have noticeable bias. Instead using "the number of testing errors", the measure of APP is more reliable under this scenario. In this case, it's easy to see in Table 2 that, the APP of the rigorous 3-fold CV is very consistent to that of the independent testing, whereas the "loose" LOOCV is not. This gives a good example on how a "loose" LOOCV brings in the so-called "gene-selection bias".

#### Small round blue-cell tumor (SRBCT) data

The SRBCT data was originally published by Khan et al. [27]. The tumor types include Ewing family of tumors (EWS), rhabdomyosarcoma (RMS), neuroblastoma (NB) and non-Hodgkin lymphoma (NHL). The dataset of the four tumor types is composed of 2308 genes and 63 samples, while 25 blinded testing samples are available. In this study, we only focused on two classes, EWS and NB. Thus, there are only 35 training sample (23 EWS and 12 NB) and 12 testing samples (6 EWS and 6 NB).

**Figure 7:**
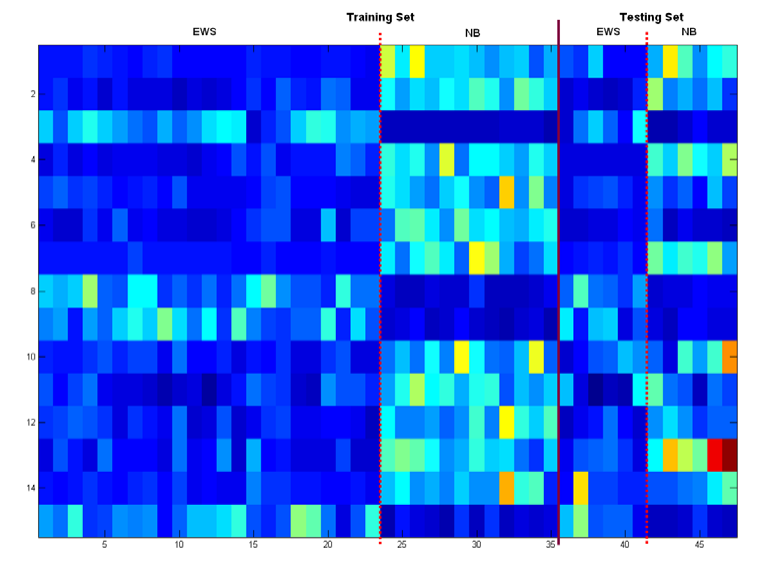
The heat map of the gene expression levels of the 15 found significant genes for the SRBCT dataset. All the legends are same as those in Fig. 6 except that the two classes are EWS and NB.

We applied the same procedure as we did in the leukemia data case to this dataset. The computational settings were also almost the same except that *π*_*j *_for all *j *was set at 0.003. The overall performance report is given in Table 4. The KIGP with the PK(1) performed best with respect to both the independent testing and the rigorous 3-fold CV. The 15 significant genes found by the KIGP with the PK(1) are listed in Table 5. The NLF plot is shown in Fig. 8b. The heat map of the significant genes for all samples is drawn in Fig. 7. The posterior PDF of the width parameter of the GK is depicted in Fig. 9b. In Table 5, the genes with asterisks (gene indices 976, 823, 842, 437 and 1700) are those not reported by the original paper [27]. Based on the heat map plot (Fig. 7), except the gene 823, the other 4 genes (gene indices 976, 842, 437, and 1700) are consistent through the training samples to the testing samples.

**Table 4:** Summary of the results from applying the proposed KIGP to the SRBCT dataset

**Performance Measure**	**Test**				**CV (3-fold)**			**LOOCV (fixed genes)**		
	
	**ANN**	**PK**	**GK**	**LK**	**PK**	**GK**	**LK**	**PK**	**GK**	**LK**
**ERR #**	0/12	0/12	0/12	0/12	0/35	2/35	0/35	0/35	0/35	0/35
**APP**	0.923	0.945	0.781	0.865	0.875	0.794	0.823	0.998	0.909	0.997

All the captions are same as in Table 2.

"ANN" stands for the "artificial neural network" method used by the paper [27]

**Table 5:** Summary of the genes found by applying the KIGP with PK(1) to the SRBCT dataset

**Index**	**NLF**	**Image ID**	**Gene Description**
255	11.36	325182	cadherin 2, N-cadherin (neuronal)
976*	10.88	786084	chromobox homolog 1 (Drosophila HP1 beta)
1389	10.19	770394	Fc fragment of IgG, receptor, transporter, alpha
742	9.28	812105	transmembrane protein
2144	8.12	308231	Homo sapiens incomplete cDNA for a mutated allele of a myosin class I, myh-1c
823*	7.53	134748	glycine cleavage system protein H (aminomethyl carrier)
2050	6.61	295985	ESTs
842*	6.08	810057	cold shock domain protein A
545	5.27	1435862	antigen identified by monoclonal antibodies 12E7, F21 and O13
867	5.22	784593	ESTs
481	5.15	825411	N-acetylglucosamine receptor 1 (thyroid)
1662	4.82	377048	Homo sapiens incomplete cDNA for a mutated allele of a myosin class I, myh-1c
1601	4.81	629896	microtubule-associated protein 1B
437*	4.42	448386	
1700*	4.20	796475	ESTs, Moderately similar to skeletal muscle LIM-protein FHL3 [H. sapiens]

*: Index of the Genes not reported in[27].

Similar to the Leukemia data case, the APP of the rigorous 3-fold CV is very consistent to that of the independent testing while the "loose" LOOCV is rather biased. We also found that the KIGP with the PK(1) outperformed the Artificial Neural Network (ANN, [27]) method in terms of APP (and both methods gave 0 testing errors).

#### Breast cancer data

The hereditary breast cancer data used in this example was published by Hedenfalk et al. [28], in which cDNA microarrays were used in conjunction with classification algorithms to show the feasibility of using the differences in global gene expression profiles to separate BRCA1 and BRCA2/sporadic. 22 breast cancer tumors were examined: 7 with BRCA1, 8 with BRCA2 and 7 considered sporadic. 3226 genes were investigated for each sample. We labeled the samples with BRCA1 as the class "1" and others as the class "-1".

The computational procedure and settings of this example are same as those in the SRBCT case except that there is no independent testing. In order to highlight the "gene-selection bias" problem, besides running a rigorous 3-fold CV procedure to measure the performance of a kernel type, we further added a "loose" 3-fold CV procedure (like the "loose" LOOCV, the CV was only run in the "prediction phase"). The overall performance report is provided in Table 6. Based on the rigorous 3-fold CV, we selected the GK(3.19) as the fitted kernel for this dataset. The posterior PDF of the width parameter is shown in Fig. 9c. We list the 9 significant genes found by the GK(3.19) in Table 7. There are two genes (gene 1851 and gene 2893 marked with asterisks in Table 7) that were not reported by the original paper [28]. The NLF plot is shown in Fig. 8c.

**Table 6:** Summary of the results from applying the KIGP to the breast cancer dataset

**Performance Measure**	**CV (3-fold)**			**CV (3-fold, fixed genes)**			**LOOCV (fixed genes)**		
	
	**PK**	**GK**	**LK**	**PK**	**GK**	**LK**	**PK**	**GK**	**LK**
**ERR #**	4/22	3/22	5/22	0/22	0/22	0/22	0/22	0/22	0/22
**APP**	0.685	0.739	0.662	0.855	0.878	0.929	0.903	0.889	0.995

All the captions are same as in Table 2, except that there is no independent testing.

The columns labeled by "CV (3-fold, fixed genes)" are for the loose 3-fold CVs (each CV only involves the "prediction phase" of an KIGP).

**Table 7:** Summary of the genes found by applying the KIGP with GK(3.19) to the breast cancer dataset

**Index**	**NLF**	**Clone ID**	**Gene Description**
1999	4.44	247818	ESTs
2734	4.21	46019	minichromosome maintenance deficient (S. cerevisiae) 7
1851*	4.06	293977	ESTs
585	3.89	293104	phytanoyl-CoA hydroxylase (Refsum disease)
2423	3.85	26082	very low density lipoprotein receptor
1443	3.85	566887	chromobox homolog 3 (Drosophila HP1 gamma)
2893*	3.81	32790	mutS (E. coli) homolog 2 (colon cancer, nonpolyposis type 1)
1068	3.81	840702	SELENOPHOSPHATE SYNTHETASE ; Human selenium donor protein
1008	3.81	897781	keratin 8

*: Index of the Genes not reported in[28].

It's not surprising to find that the general performance of the KIGP with an LK or the PK(1) was not good since we notice that there is an unusual local peak on the left side of the posterior PDF of the width parameter r (Fig. 9c). This local peak usually implies the existence of non-linearity in the data for the given problem. A fairly logical reason for this phenomena can be found in [29], in which Efron showed that the empirical null of this dataset was significantly different from its theoretical null based on a large-scale simultaneous 2-sample t-test and he argued that this was probably due to the fact that the experimental methodology used in the original paper had induced substantial correlations among the various microarrays.

This example is also a good case to show the "gene-selection bias" problem. In Table 6, with the selected significant genes found by training all the available samples, the performance of the KIGP with an LK from the "loose" 3-fold CV was much better than that of the KIGP with a GK. However, from the results of the rigorous 3-fold CV, the KIGP with an LK gave very poor predictive performance, while the KIGP with an GK still worked reasonably well.

#### Colon data

This dataset was originally published by Alon et al. [30] and we noticed that Dettling [16] has reported the performances of many state-of-the-art learning methods that had been applied to this dataset. We applied the KIGP method to this dataset so as to have a more side-by-side performance comparison with other methods. [16] did a pre-filtering of genes based on the Wilcoxon test statistic and only ran all the simulations within a 200-gene pool. However, based on the reported procedure, it should not bring in much gene-selection bias. Therefore, it forms a good dataset for comparing different microarray data analysis methods.

We applied the proposed KIGP to the whole dataset without any pre-filtering to preclude any possible gene-selection bias. The computational procedure and settings of this example are very similar to those in the SRBCT case except that there is no independent testing. As for the cross validation procedure, we ran 5 independent simulations and reported the average of the results to decrease the possible data split bias. The procedure for the data splitting is described in the "Kernel type competition" subsection. We did this with each kernel type: PK, GK and LK, respectively. The resulted performance and the performances of other methods (reported by [16]) are summarized in Table 8. We found that the MR of the KIGP with an GK is very close to that of the best classifier (PAM in this case) shown in the list. It is worth mentioning that PAM's performances were ranked as average to significantly worse than 6 other methods, especially comparing to kernel-induced methods such as the SVM for other published real datasets (such as the leukemia dataset, the prostate dataset and the lymphoma dataset [16]). The KIGP method with an appropriate kernel is at least not worse than the SVM.

**Table 8:** Summary of the performance comparison on applying different classifiers to the colon dataset

**Classifier**	**MR**
KIGP(PK)	0.166
KIGP(GK)	0.129
KIGP(LK)	0.198
BagBoost	0.161
Boosting	0.191
RF	0.149
SVM	0.151
PAM	0.119
DLDA	0.129
kNN	0.164

For the KIGP with each of the three different kernel types (PK, GK, LK), we took 5 independent rigorous 3-fold CVs to 62 samples (each CV involves all 3 phases of an KIGP) and reported the average MR. For the 7 referred classifiers, the results and the experimental details were originally reported by[16].

RF: "Random Forests"

PAM: "Nearest shrunken centroids"

DLDA: "diagonal linear discriminant analysis"

Based on the MR of the rigorous 3-fold CV, we selected the GK as the winning kernel type. We then ran KIGP with a GK to all the available samples. After the "kernel parameter fitting phase", with the posterior PDF of the width parameter (Fig. 9d), we fixed the kernel as the GK(2.38). The resulted NLF plot with the GK(2.38) after the "gene selection phase" is depicted in Fig. 8d. The indices of the 8 identified significant genes are provided in Table 9.

**Table 9:** Summary of the genes found by applying the KIGP with GK(2.38) to the colon dataset

**Index**	**NLF**
377	6.54
493	6.25
249	5.48
267	4.51
245	4.34
765	4.29
513	3.94
14	3.88

Another interesting finding of this experiment is that, based on the results of the "loose" CV, the KIGP/LK performed better than the KIGP/GK for this dataset. However, with a multiple rigorous 3-fold CV, it turned out that KIGP/GK was the more reliable kernel type for this problem. When we checked the heat map of the significant gene set identified by the KIGP/GK (Table 9), we found that a few samples, particularly including the sample #18, #20, #45, #49 and #56, are significantly different from other samples in their labeled class. However, they are very consistent to those samples in their opposite class. In fact, these samples were also almost always misclassified by the KIGP in the multiple rigorous 3-fold CV tests. We therefore suspect that these samples are mistakenly phenotyped. We think that this is probably the reason why all other learning methods referred in Table 8 do not perform well for this colon dataset. This also supports the nature of a KIGP/GK being less sensitive to the mislabeled training samples than a KIGP/LK.

## Discussions and Conclusion

This work was motivated by the data analysis challenges posed by microarray gene expression experiments and the mathematical beauty of the kernel-imbedding approach in their ability to solve a non-linear classification problem in the feature space rather than in the observation space. We have presented a unified supervised learning model named kernel-imbedded Gaussian process (KIGP) under a hierarchical Bayesian framework. This model was specifically designed for automatic learning and profiling of microarray gene expression patterns. In the simulated examples, without knowing anything of the underlying generative model, the proposed KIGP method has been shown to perform very close to the Bayesian bound not only in the linear case, but also in the non-linear case.

With a probit regression setting and the introduction of latent variables, the KIGP model was set for a binary disease classification problem. An algorithm with a cascading structure was proposed to solve this problem and a Gibbs sampler was built as the mechanical core to do the Bayesian inferences. Given a kernel type such as a Gaussian kernel or a polynomial kernel, with the training data as input, the fitted parameter(s) of the kernel type and a set of significant genes will be the output of the algorithm. The algorithm also offers a probabilistic class prediction for each sample. The proposed KIGP can explore not only the linear but also the potential non-linear relationship between the target disease and its associated explanatory genes. Comparing to the regular SVM (a very popular kernel-induced learning method), the proposed KIGP has two advantages. First, the probabilistic class prediction from the KIGP could be insightful for borderline cases in real-world applications. Second, the KIGP method has implemented specific procedure for tuning the kernel parameter(s) (such as the width parameter of a Gaussian kernel or the degree parameter of a polynomial kernel) and the model parameters (such as the variance of the noise term). Tuning parameters has always been one of the key issues for non-linear parametric learning methods. The results of the simulated examples show that the KIGP significantly outperformed the regular SVM method with RFE as a gene selection strategy in a non-linear case and it provided more useful information, such as the posterior PDF of the parameters, for further prediction and analysis as well. Computationally, KIGP is also proven to be robust, therefore it's very amenable to be adopted to a Gibbs sampling system. Both the simulated examples and the real data studies have demonstrated the effectiveness of the proposed method.

There are still a few interesting problems left for future research. For example, although the KIGP in this study is developed to only solve a binary classification problem, it can easily be extended to a multi-class classification problem based on a multinomial probit regression setting. On the other hand, some other problems are not only challenging but also critical. First, the kernel type competing problem is still a tough issue. The use of the predictive fit measure method discussed in the "Methods" section is simple to formulate, but it may be problematic when the independent testing set is not available and/or there are many candidate kernel types. We are currently working on addressing this issue by implementing a reversible jump Markov Chain Monte Carlo (RJMCMC) algorithm as a simultaneous integrative approach for kernel type selection within the KIGP framework. Another important problem is the independent prior assumption on elements of the gene-selection vector **γ **and the "component-wise drawing" strategy to sample it. Although this will eventually lead to convergence based on the MCMC theory, it may take a very long time if the true underlying explanatory genes are highly correlated with each other. Therefore, a proper kernel-induced clustering algorithm under some proper generative model will definitely be helpful on this regard. Furthermore, if a more appropriate prior for **γ **can be found, the dependency between genes can be simply taken into account to the whole framework by sampling **γ **not in a component-wise fashion but in a block-wise fashion instead. This will then dramatically increase the speed for reaching convergence.

Interestingly, building a kernel based on the feature of the given data and the classification problem is the ideal way to take full advantage of the kernel-induced learning algorithm. For example, if an appropriate generative model is available for the given dataset, a class of kernels named "natural kernels" is applicable in this context. This problem and the pre-clustering problem mentioned above seemingly share many fundamental elements. However, the further investigation of this is beyond the scope of this paper.

## Methods

### Problem formulation

We consider a binary classification problem. Suppose there are *n *training samples and let **y **= [*y*_1_, *y*_2_,...,*y*_*n*_]' denote the class labels, where *y*_*i *_= 1 indicates the sample *i *being in the class "I" and *y*_*i *_= -1 indicates it being in the other class (i.e. not class "I"), for *i *= 1,2,...,*n*. For each sample, there are *p *genes being investigated and we define the gene expression matrix **X **as

X=[Gene 1Gene 2…Gene pX11X12…X1p⋮⋮⋱⋮Xn1Xn2⋯Xnp]     (1)
 MathType@MTEF@5@5@+=feaafiart1ev1aaatCvAUfKttLearuWrP9MDH5MBPbIqV92AaeXatLxBI9gBaebbnrfifHhDYfgasaacH8akY=wiFfYdH8Gipec8Eeeu0xXdbba9frFj0=OqFfea0dXdd9vqai=hGuQ8kuc9pgc9s8qqaq=dirpe0xb9q8qiLsFr0=vr0=vr0dc8meaabaqaciaacaGaaeqabaqabeGadaaakeaaieqacqWFybawcqGH9aqpdaWadaqaauaabeqaeqaaaaaabaGaee4raCKaeeyzauMaeeOBa4MaeeyzauMaeeiiaaIaeGymaedabaGaee4raCKaeeyzauMaeeOBa4MaeeyzauMaeeiiaaIaeGOmaidabaGaeSOjGSeabaGaee4raCKaeeyzauMaeeOBa4MaeeyzauMaeeiiaaIaemiCaahabaGaemiwaG1aaSbaaSqaaiabigdaXiabigdaXaqabaaakeaacqWGybawdaWgaaWcbaGaeGymaeJaeGOmaidabeaaaOqaaiablAcilbqaaiabdIfaynaaBaaaleaacqaIXaqmcqWGWbaCaeqaaaGcbaGaeSO7I0eabaGaeSO7I0eabaGaeSy8I8eabaGaeSO7I0eabaGaemiwaG1aaSbaaSqaaiabd6gaUjabigdaXaqabaaakeaacqWGybawdaWgaaWcbaGaemOBa4MaeGOmaidabeaaaOqaaiabl+UimbqaaiabdIfaynaaBaaaleaacqWGUbGBcqWGWbaCaeqaaaaaaOGaay5waiaaw2faaiaaxMaacaWLjaWaaeWaaeaacqaIXaqmaiaawIcacaGLPaaaaaa@6BCC@

The data matrix **X **usually should be normalized for each gene (each column of **X**). In order to handle the gene selection problem, we further define the gene-selection vector **γ **as:

γ=[γ1,γ2,...,γp]′, where γj={1if the jth gene is selected0otherwise,j=1, ..p     (2)
 MathType@MTEF@5@5@+=feaafiart1ev1aaatCvAUfKttLearuWrP9MDH5MBPbIqV92AaeXatLxBI9gBaebbnrfifHhDYfgasaacH8akY=wiFfYdH8Gipec8Eeeu0xXdbba9frFj0=OqFfea0dXdd9vqai=hGuQ8kuc9pgc9s8qqaq=dirpe0xb9q8qiLsFr0=vr0=vr0dc8meaabaqaciaacaGaaeqabaqabeGadaaakeaaiiqacqWFZoWzcqGH9aqpcqGGBbWwiiGacqGFZoWzdaWgaaWcbaGaeGymaedabeaakiabcYcaSiab+n7aNnaaBaaaleaacqaIYaGmaeqaaOGaeiilaWIaeiOla4IaeiOla4IaeiOla4IaeiilaWIae43SdC2aaSbaaSqaaiabdchaWbqabaGccqGGDbqxdaahaaWcbeqaaOGamai0gkdiIcaacqGGSaalcqqGGaaicqqG3bWDcqqGObaAcqqGLbqzcqqGYbGCcqqGLbqzcqqGGaaicqGFZoWzdaWgaaWcbaGaemOAaOgabeaakiabg2da9maaceqabaqbaeqabiGaaaqaaiabigdaXaqaaiabbMgaPjabbAgaMjabbccaGiabbsha0jabbIgaOjabbwgaLjabbccaGiabbQgaQjabbsha0jabbIgaOjabbccaGiabbEgaNjabbwgaLjabb6gaUjabbwgaLjabbccaGiabbMgaPjabbohaZjabbccaGiabbohaZjabbwgaLjabbYgaSjabbwgaLjabbogaJjabbsha0jabbwgaLjabbsgaKbqaaiabicdaWaqaaiabb+gaVjabbsha0jabbIgaOjabbwgaLjabbkhaYjabbEha3jabbMgaPjabbohaZjabbwgaLbaacqGGSaalcqWGQbGAcqGH9aqpcqaIXaqmcqGGSaalcqqGGaaicqGGUaGlcqGGUaGlcqWGWbaCaiaawUhaaiaaxMaacaWLjaWaaeWaaeaacqaIYaGmaiaawIcacaGLPaaaaaa@8E8E@

**X**_**γ **_is defined as the gene expression matrix corresponding to the selected genes in accordance to the gene-selection vector **γ**. I.e.

Xγ=[Xγ,11,Xγ,12,...,Xγ,1q⋮Xγ,n1,Xγ,n2,...,Xγ,nq]=[xγ1⋮xγn],     (3)
 MathType@MTEF@5@5@+=feaafiart1ev1aaatCvAUfKttLearuWrP9MDH5MBPbIqV92AaeXatLxBI9gBaebbnrfifHhDYfgasaacH8akY=wiFfYdH8Gipec8Eeeu0xXdbba9frFj0=OqFfea0dXdd9vqai=hGuQ8kuc9pgc9s8qqaq=dirpe0xb9q8qiLsFr0=vr0=vr0dc8meaabaqaciaacaGaaeqabaqabeGadaaakeaaieqacqWFybawdaWgaaWcbaacciGae43SdCgabeaakiabg2da9maadmaabaqbaeaabmqaaaqaaiabdIfaynaaBaaaleaacqGFZoWzcqGGSaalcqaIXaqmcqaIXaqmaeqaaOGaeiilaWIaemiwaG1aaSbaaSqaaiab+n7aNjabcYcaSiabigdaXiabikdaYaqabaGccqGGSaalcqGGUaGlcqGGUaGlcqGGUaGlcqGGSaalcqWGybawdaWgaaWcbaGae43SdCMaeiilaWIaeGymaeJaemyCaehabeaaaOqaaiabl6UinbqaaiabdIfaynaaBaaaleaacqGFZoWzcqGGSaalcqWGUbGBcqaIXaqmaeqaaOGaeiilaWIaemiwaG1aaSbaaSqaaiab+n7aNjabcYcaSiabd6gaUjabikdaYaqabaGccqGGSaalcqGGUaGlcqGGUaGlcqGGUaGlcqGGSaalcqWGybawdaWgaaWcbaGae43SdCMaeiilaWIaemOBa4MaemyCaehabeaaaaaakiaawUfacaGLDbaacqGH9aqpdaWadaqaauaabaqadeaaaeaacqWF4baEdaWgaaWcbaGae43SdCMaeGymaedabeaaaOqaaiabl6Uinbqaaiab=Hha4naaBaaaleaacqGFZoWzcqWGUbGBaeqaaaaaaOGaay5waiaaw2faaiabcYcaSiaaxMaacaWLjaWaaeWaaeaacqaIZaWmaiaawIcacaGLPaaaaaa@7715@

where the *j *th column of **X**_**γ **_is the *i *th column of the matrix **X **while the index of the *j *th non-zero element in the vector **γ **is *i*. In formula (3), there are *q *genes being selected out from the total *p *genes; and *q *<<*p *in a typical gene selection problem. Formulating the problem in a regression setting, we introduce *n *latent variables *z*_1_, *z*_2_,..., *z*_*n*_, such that

*z*_*i *_= *g*(**X**_**γ***i*_) + *b *+ *e*_*i *_= *t*_*i *_+ *b *+ *e*_*i*_, and

yi={1if zi≥0−1if zi<0,i=1,2,...,n,     (4)
 MathType@MTEF@5@5@+=feaafiart1ev1aaatCvAUfKttLearuWrP9MDH5MBPbIqV92AaeXatLxBI9gBaebbnrfifHhDYfgasaacH8akY=wiFfYdH8Gipec8Eeeu0xXdbba9frFj0=OqFfea0dXdd9vqai=hGuQ8kuc9pgc9s8qqaq=dirpe0xb9q8qiLsFr0=vr0=vr0dc8meaabaqaciaacaGaaeqabaqabeGadaaakeaacqWG5bqEdaWgaaWcbaGaemyAaKgabeaakiabg2da9maaceqabaqbaeqabiGaaaqaaiabigdaXaqaaiabbMgaPjabbAgaMjabbccaGiabdQha6naaBaaaleaacqWGPbqAaeqaaOGaeyyzImRaeGimaadabaGaeyOeI0IaeGymaedabaGaeeyAaKMaeeOzayMaeeiiaaIaemOEaO3aaSbaaSqaaiabdMgaPbqabaGccqGH8aapcqaIWaamaaGaeiilaWIaemyAaKMaeyypa0JaeGymaeJaeiilaWIaeGOmaiJaeiilaWIaeiOla4IaeiOla4IaeiOla4IaeiilaWIaemOBa4gacaGL7baacqGGSaalcaWLjaGaaCzcamaabmaabaGaeGinaqdacaGLOaGaayzkaaaaaa@56D5@

where **x**_*γi *_denotes the *i *th row of the matrix **X**_*γ*_; *e*_*i *_presents the independent noise term, which is assumed to be Guassian distributed with zero mean, *σ*^2 ^variance; *b *is the intercept term; and the link function *g*(·) is assumed to be chosen from a class of real-valued functions and the output of which is a Gaussian process. In the vector form, we define **z **= [*z*_1_, *z*_2_,..., *z*_*n*_]', **t **= [*t*_1_, *t*_2_,..., *t*_*n*_]' and **e **= [*e*_1_, *e*_2_,..., *e*_*n*_]'. Note that, if *g*(·) is restricted to a linear function and *σ*^2 ^is fixed at 1, model (4) is very similar to a linear probit regression setting.

### Kernel-Imbedded Gaussian Processes (KIGPs)

In general, a continuous stochastic process is a collection of random variables, and each of these random variables takes on real values from a probability distribution function. If we consider the outputs of a learning function *g*(·), where *g *is chosen according to some distribution *D *defined over a class of real-valued functions, then the collection of these outputs is also a stochastic process and the distribution *D *presents the prior belief in the likelihood.

A Gaussian process is a continuous stochastic process such that the marginal distribution for any finite subset of the collection of its outputs is a zero mean Gaussian distribution. In this paper, as defined in formula (4), *t*_*i *_= *g*(**x**_*γi*_), where **x**_**γi **_= [*x*_*γ,i*1_, *x*_*γ,i*2_,..., *x*_*γ,iq*_], *i *= 1,2,..., *n*; and in the formula, we assume

*P*_*g*~*D*_([*g*(**x**_**γ1**_), *g*(**x**_**γ2**_),..., *g*(**x**_**γn**_)] = [*t*_1_, *t*_2_,..., *t*_*n*_]) ∝ exp⁡(−12t'K−1t)
 MathType@MTEF@5@5@+=feaafiart1ev1aaatCvAUfKttLearuWrP9MDH5MBPbIqV92AaeXatLxBI9gBaebbnrfifHhDYfgasaacH8akY=wiFfYdH8Gipec8Eeeu0xXdbba9frFj0=OqFfea0dXdd9vqai=hGuQ8kuc9pgc9s8qqaq=dirpe0xb9q8qiLsFr0=vr0=vr0dc8meaabaqaciaacaGaaeqabaqabeGadaaakeaacyGGLbqzcqGG4baEcqGGWbaCcqGGOaakcqGHsisldaWcaaqaaiabigdaXaqaaiabikdaYaaaieqacqWF0baDcqGGNaWjcqWFlbWsdaahaaWcbeqaaiabgkHiTiabigdaXaaakiab=rha0jabcMcaPaaa@3C5A@, where

**K**_*ij *_= *K*(**x**_**γi**_, **x**_**γj**_), *i*, *j *= 1,2,..., *n*.     (5)

In (5), *K*(**x**_**γi**_, **x**_**γj**_) is a function defined in the observation space and it conceptually represents the inner product for sample vectors **x**_**γi **_and **x**_**γj **_in the feature space, ⟨Ψ(**x**_**γi**_), Ψ(**x**_**γj**_)⟩ (assuming Ψ(·) is the mapping function from the observation space to the feature space). **K **is a kernel matrix called the Mercer kernel. Formula (5) formulates our prior belief for the learning model and the kernel function *K*(·,·) uniquely decides the properties of our learning functions. Some of the most commonly used kernel functions include:

Linear kernel: *K*(**x**_*γ***i**_, **x**_*γ***j**_) = ⟨**x**_*γ***i**_, **x**_*γ***j**_⟩     (6a)

Polynomial kernel: *K*(**x**_*γ***i**_, **x**_*γ***j**_) = (⟨**x**_*γ***i**_, **x**_*γ***j**_⟩ + 1)^*d*^, where *d *= 1,2,.. is the degree parameter.     (6b)

Gaussian kernel: *K*(**x**_*γ***i**_, **x**_*γ***j**_) = exp⁡(−‖xγi−xγj‖22r2)
 MathType@MTEF@5@5@+=feaafiart1ev1aaatCvAUfKttLearuWrP9MDH5MBPbIqV92AaeXatLxBI9gBaebbnrfifHhDYfgasaacH8akY=wiFfYdH8Gipec8Eeeu0xXdbba9frFj0=OqFfea0dXdd9vqai=hGuQ8kuc9pgc9s8qqaq=dirpe0xb9q8qiLsFr0=vr0=vr0dc8meaabaqaciaacaGaaeqabaqabeGadaaakeaacyGGLbqzcqGG4baEcqGGWbaCcqGGOaakcqGHsisldaWcaaqaamaafmaabaacbeGae8hEaG3aaSbaaSqaaGGaciab+n7aNjab=LgaPbqabaGccqGHsislcqWF4baEdaWgaaWcbaGae43SdCMae8NAaOgabeaaaOGaayzcSlaawQa7amaaCaaaleqabaGaeGOmaidaaaGcbaGaeGOmaiJaemOCai3aaWbaaSqabeaacqaIYaGmaaaaaOGaeiykaKcaaa@45B2@, where *r *> 0 is the width parameter.     (6c)

In (6a) and (6b), the term ⟨**x**_*γ***i**_, **x**_*γ***j**_⟩ presents the inner product between the vectors **x**_*γi *_and **x**_*γj*_. When one uses the linear kernel, the feature space is the same as the observation space. In this paper, we refer the linear kernel as the LK, the polynomial kernel with degree "d" as the PK(d) and the Gaussian kernel with width "r" as the GK(r). We primarily focus on the KIGP method with the Gaussian kernel and the polynomial kernel, and discuss them in parallel.

In model (4), we have the latent vector **z **= **t **+ **e **+ *b***1_n_**, where **e **~ *N*(**0**, *σ*^2^**I_n_**), **I**_**n **_denotes the *n *× *n *identity matrix, and **1_n _**presents the *n *× 1 vector with all the elements being equal to 1; *N*(·,·) denotes the multivariate normal distribution. Hence,

*P*(**z**|**t**) ∝ exp(-12
 MathType@MTEF@5@5@+=feaafiart1ev1aaatCvAUfKttLearuWrP9MDH5MBPbIqV92AaeXatLxBI9gBaebbnrfifHhDYfgasaacH8akY=wiFfYdH8Gipec8Eeeu0xXdbba9frFj0=OqFfea0dXdd9vqai=hGuQ8kuc9pgc9s8qqaq=dirpe0xb9q8qiLsFr0=vr0=vr0dc8meaabaqaciaacaGaaeqabaqabeGadaaakeaadaWcaaqaaiabigdaXaqaaiabikdaYaaaaaa@2E9E@(**z **- **t **- *b***1_n_**)'Ω^-1^(**z **- **t **- *b***1_n_**)), where Ω = *σ*^2^**I_n_**.     (7)

With the Bayes rule, we have

P(t˜,t|x˜γ,Xγ,z)=P(z|t,x˜γ,Xγ)P(t˜,t|x˜γ,Xγ)P(z|x˜γ,Xγ)∝P(z|t)P(t˜,t|x˜γ,Xγ),     (8)
 MathType@MTEF@5@5@+=feaafiart1ev1aaatCvAUfKttLearuWrP9MDH5MBPbIqV92AaeXatLxBI9gBaebbnrfifHhDYfgasaacH8akY=wiFfYdH8Gipec8Eeeu0xXdbba9frFj0=OqFfea0dXdd9vqai=hGuQ8kuc9pgc9s8qqaq=dirpe0xb9q8qiLsFr0=vr0=vr0dc8meaabaqaciaacaGaaeqabaqabeGadaaakeaacqWGqbaucqGGOaakcuWG0baDgaacaiabcYcaSGqabiab=rha0jabcYha8jqb=Hha4zaaiaWaaSbaaSqaaGGaciab+n7aNbqabaGccqGGSaalcqWFybawdaWgaaWcbaGae43SdCgabeaakiabcYcaSiab=Pha6jabcMcaPiabg2da9maalaaabaGaemiuaaLaeiikaGIae8NEaONaeiiFaWNae8hDaqNaeiilaWIaf8hEaGNbaGaadaWgaaWcbaGae43SdCgabeaakiabcYcaSiab=HfaynaaBaaaleaacqGFZoWzaeqaaOGaeiykaKIaemiuaaLaeiikaGIafmiDaqNbaGaacqGGSaalcqWF0baDcqGG8baFcuWF4baEgaacamaaBaaaleaacqGFZoWzaeqaaOGaeiilaWIae8hwaG1aaSbaaSqaaiab+n7aNbqabaGccqGGPaqkaeaacqWGqbaucqGGOaakcqWF6bGEcqGG8baFcuWF4baEgaacamaaBaaaleaacqGFZoWzaeqaaOGaeiilaWIae8hwaG1aaSbaaSqaaiab+n7aNbqabaGccqGGPaqkaaGaeyyhIuRaemiuaaLaeiikaGIae8NEaONaeiiFaWNae8hDaqNaeiykaKIaemiuaaLaeiikaGIafmiDaqNbaGaacqGGSaalcqWF0baDcqGG8baFcuWF4baEgaacamaaBaaaleaacqGFZoWzaeqaaOGaeiilaWIae8hwaG1aaSbaaSqaaiab+n7aNbqabaGccqGGPaqkcqGGSaalcaWLjaGaaCzcamaabmaabaGaeGioaGdacaGLOaGaayzkaaaaaa@886D@

where x˜
 MathType@MTEF@5@5@+=feaafiart1ev1aaatCvAUfKttLearuWrP9MDH5MBPbIqV92AaeXatLxBI9gBaebbnrfifHhDYfgasaacH8akY=wiFfYdH8Gipec8Eeeu0xXdbba9frFj0=OqFfea0dXdd9vqai=hGuQ8kuc9pgc9s8qqaq=dirpe0xb9q8qiLsFr0=vr0=vr0dc8meaabaqaciaacaGaaeqabaqabeGadaaakeaaieqacuWF4baEgaacaaaa@2E3A@_*γ *_is the new predictor associated with the given gene-selection vector **γ **and t˜
 MathType@MTEF@5@5@+=feaafiart1ev1aaatCvAUfKttLearuWrP9MDH5MBPbIqV92AaeXatLxBI9gBaebbnrfifHhDYfgasaacH8akY=wiFfYdH8Gipec8Eeeu0xXdbba9frFj0=OqFfea0dXdd9vqai=hGuQ8kuc9pgc9s8qqaq=dirpe0xb9q8qiLsFr0=vr0=vr0dc8meaabaqaciaacaGaaeqabaqabeGadaaakeaacuWG0baDgaacaaaa@2E2C@ is the posterior output (without intercept *b*) with respect to x˜
 MathType@MTEF@5@5@+=feaafiart1ev1aaatCvAUfKttLearuWrP9MDH5MBPbIqV92AaeXatLxBI9gBaebbnrfifHhDYfgasaacH8akY=wiFfYdH8Gipec8Eeeu0xXdbba9frFj0=OqFfea0dXdd9vqai=hGuQ8kuc9pgc9s8qqaq=dirpe0xb9q8qiLsFr0=vr0=vr0dc8meaabaqaciaacaGaaeqabaqabeGadaaakeaaieqacuWF4baEgaacaaaa@2E3A@_*γ*_, provided the matrix **X**_*γ *_and the latent output **z**. With a kernel such as defined by (6) and assuming an intercept *b *and a variance of noise *σ*^2 ^are both given, plugging (5) and (7) into (8) and integrating out **t**, the marginal distribution of t˜
 MathType@MTEF@5@5@+=feaafiart1ev1aaatCvAUfKttLearuWrP9MDH5MBPbIqV92AaeXatLxBI9gBaebbnrfifHhDYfgasaacH8akY=wiFfYdH8Gipec8Eeeu0xXdbba9frFj0=OqFfea0dXdd9vqai=hGuQ8kuc9pgc9s8qqaq=dirpe0xb9q8qiLsFr0=vr0=vr0dc8meaabaqaciaacaGaaeqabaqabeGadaaakeaacuWG0baDgaacaaaa@2E2C@ given x˜
 MathType@MTEF@5@5@+=feaafiart1ev1aaatCvAUfKttLearuWrP9MDH5MBPbIqV92AaeXatLxBI9gBaebbnrfifHhDYfgasaacH8akY=wiFfYdH8Gipec8Eeeu0xXdbba9frFj0=OqFfea0dXdd9vqai=hGuQ8kuc9pgc9s8qqaq=dirpe0xb9q8qiLsFr0=vr0=vr0dc8meaabaqaciaacaGaaeqabaqabeGadaaakeaaieqacuWF4baEgaacaaaa@2E3A@_*γ*_, **X**_*γ *_and **z **yields a Gaussian distribution as follow [26]:

t˜
 MathType@MTEF@5@5@+=feaafiart1ev1aaatCvAUfKttLearuWrP9MDH5MBPbIqV92AaeXatLxBI9gBaebbnrfifHhDYfgasaacH8akY=wiFfYdH8Gipec8Eeeu0xXdbba9frFj0=OqFfea0dXdd9vqai=hGuQ8kuc9pgc9s8qqaq=dirpe0xb9q8qiLsFr0=vr0=vr0dc8meaabaqaciaacaGaaeqabaqabeGadaaakeaacuWG0baDgaacaaaa@2E2C@|x˜
 MathType@MTEF@5@5@+=feaafiart1ev1aaatCvAUfKttLearuWrP9MDH5MBPbIqV92AaeXatLxBI9gBaebbnrfifHhDYfgasaacH8akY=wiFfYdH8Gipec8Eeeu0xXdbba9frFj0=OqFfea0dXdd9vqai=hGuQ8kuc9pgc9s8qqaq=dirpe0xb9q8qiLsFr0=vr0=vr0dc8meaabaqaciaacaGaaeqabaqabeGadaaakeaaieqacuWF4baEgaacaaaa@2E3A@_*γ*_, **X**_*γ*_, **z **~ *N*(*f*(x˜
 MathType@MTEF@5@5@+=feaafiart1ev1aaatCvAUfKttLearuWrP9MDH5MBPbIqV92AaeXatLxBI9gBaebbnrfifHhDYfgasaacH8akY=wiFfYdH8Gipec8Eeeu0xXdbba9frFj0=OqFfea0dXdd9vqai=hGuQ8kuc9pgc9s8qqaq=dirpe0xb9q8qiLsFr0=vr0=vr0dc8meaabaqaciaacaGaaeqabaqabeGadaaakeaaieqacuWF4baEgaacaaaa@2E3A@_*γ*_, **X**_*γ*_, **z**), *V*(x˜
 MathType@MTEF@5@5@+=feaafiart1ev1aaatCvAUfKttLearuWrP9MDH5MBPbIqV92AaeXatLxBI9gBaebbnrfifHhDYfgasaacH8akY=wiFfYdH8Gipec8Eeeu0xXdbba9frFj0=OqFfea0dXdd9vqai=hGuQ8kuc9pgc9s8qqaq=dirpe0xb9q8qiLsFr0=vr0=vr0dc8meaabaqaciaacaGaaeqabaqabeGadaaakeaaieqacuWF4baEgaacaaaa@2E3A@_*γ*_, **X**_*γ*_, **z**)), where

*f*(x˜
 MathType@MTEF@5@5@+=feaafiart1ev1aaatCvAUfKttLearuWrP9MDH5MBPbIqV92AaeXatLxBI9gBaebbnrfifHhDYfgasaacH8akY=wiFfYdH8Gipec8Eeeu0xXdbba9frFj0=OqFfea0dXdd9vqai=hGuQ8kuc9pgc9s8qqaq=dirpe0xb9q8qiLsFr0=vr0=vr0dc8meaabaqaciaacaGaaeqabaqabeGadaaakeaaieqacuWF4baEgaacaaaa@2E3A@_*γ*_, **X**_*γ*_, **z**) = (**z **- *b***l_n_**)' (**K**_*γ *_+ *σ*^2^**I**)^-1 ^**k**_*γ*_, *V*(x˜
 MathType@MTEF@5@5@+=feaafiart1ev1aaatCvAUfKttLearuWrP9MDH5MBPbIqV92AaeXatLxBI9gBaebbnrfifHhDYfgasaacH8akY=wiFfYdH8Gipec8Eeeu0xXdbba9frFj0=OqFfea0dXdd9vqai=hGuQ8kuc9pgc9s8qqaq=dirpe0xb9q8qiLsFr0=vr0=vr0dc8meaabaqaciaacaGaaeqabaqabeGadaaakeaaieqacuWF4baEgaacaaaa@2E3A@_*γ*_, **X**_*γ*_, **z**) = *K*(x˜
 MathType@MTEF@5@5@+=feaafiart1ev1aaatCvAUfKttLearuWrP9MDH5MBPbIqV92AaeXatLxBI9gBaebbnrfifHhDYfgasaacH8akY=wiFfYdH8Gipec8Eeeu0xXdbba9frFj0=OqFfea0dXdd9vqai=hGuQ8kuc9pgc9s8qqaq=dirpe0xb9q8qiLsFr0=vr0=vr0dc8meaabaqaciaacaGaaeqabaqabeGadaaakeaaieqacuWF4baEgaacaaaa@2E3A@_*γ*_, x˜
 MathType@MTEF@5@5@+=feaafiart1ev1aaatCvAUfKttLearuWrP9MDH5MBPbIqV92AaeXatLxBI9gBaebbnrfifHhDYfgasaacH8akY=wiFfYdH8Gipec8Eeeu0xXdbba9frFj0=OqFfea0dXdd9vqai=hGuQ8kuc9pgc9s8qqaq=dirpe0xb9q8qiLsFr0=vr0=vr0dc8meaabaqaciaacaGaaeqabaqabeGadaaakeaaieqacuWF4baEgaacaaaa@2E3A@_*γ*_) - **k**_*γ*_'(**K**_*γ *_+ *σ*^2^**I**)^-1^**k**_*γ*_,

**K**_*γ,ij *_= *K*(**x**_*γ***i**_, **x**_*γ***j**_), **k**_*γi *_= *K*(x˜
 MathType@MTEF@5@5@+=feaafiart1ev1aaatCvAUfKttLearuWrP9MDH5MBPbIqV92AaeXatLxBI9gBaebbnrfifHhDYfgasaacH8akY=wiFfYdH8Gipec8Eeeu0xXdbba9frFj0=OqFfea0dXdd9vqai=hGuQ8kuc9pgc9s8qqaq=dirpe0xb9q8qiLsFr0=vr0=vr0dc8meaabaqaciaacaGaaeqabaqabeGadaaakeaaieqacuWF4baEgaacaaaa@2E3A@_*γ*_, **x**_*γi*_), *i*, *j *= 1,2,..., *n*.     (9)

### Supervised Microarray Data Analysis using KIGP

#### Prior specification

**(1) ***γ*_*j *_is assumed to be a priori independent for all *j*, and

Pr(*γ*_*j *_= 1) = *π*_*j*_, for *j *= 1, 2,..., *p*,     (10)

where the prior probability *π*_*j *_reflects prior knowledge of the importance of the *j*th gene.

**(2) **A non-informative prior is applied for the intercept *b*:

*P*(*b*) ∝ 1.     (11a)

This is not a proper probability distribution function (PDF), but it leads to a proper posterior PDF.

**(3) **The inverted gamma (IG) distribution is applied as the prior for the variance of noise *σ*^2^. Specifically, we assume:

*P*(*σ*^2^) ~ *IG*(1,1)     (11b)

**(4) **For the width of a Gaussian kernel (i.e. a scaling parameter), an inverted gamma distribution is also a reasonable choice as a prior. To preclude too small or too big *r *(which will make the system to be numerically unstable), we apply IG(1,1) as the prior for *r*^2^, that is

*P*(*r*^2^) ~ *IG*(1,1)     (11c)

**(5) **For the degree of a polynomial kernel, we assume a uniform distribution. In this paper, we only consider the PK(1) and the PK(2) to avoid the issue of overfitting for most practical cases. Therefore, we have *P*(*d *= 1) = *P*(*d *= 2) = 0.5.

**(6) **We assume that **γ **and *b *are a priori independent from each other, that is *P*(**γ**, *b*) = *P*(**γ**)*P*(*b*).

#### Bayesian inferences for model parameters

Based on model (4), label **y **only depends on **z**, therefore, all other model parameters are conditionally independent from **y **if **z **is given. For convenience, we drop the notation of the training set **X **in the following derivation and drop **y **as well when **z **is given. We also assume the kernel type is given and the associated kernel parameter is termed by **θ**.

**(I) Sampling from γ**|**z**, *b*, *σ*^2^, **θ**

Here, we drop the notation of the given parameters *b*, *σ*^2 ^and **θ**. With the model described in (2), (5) and (7), we have

P(z|γ)=∫tP(z|t)P(t|Xγ)dt~N(b1n,Kγ+Ω)
 MathType@MTEF@5@5@+=feaafiart1ev1aaatCvAUfKttLearuWrP9MDH5MBPbIqV92AaeXatLxBI9gBaebbnrfifHhDYfgasaacH8akY=wiFfYdH8Gipec8Eeeu0xXdbba9frFj0=OqFfea0dXdd9vqai=hGuQ8kuc9pgc9s8qqaq=dirpe0xb9q8qiLsFr0=vr0=vr0dc8meaabaqaciaacaGaaeqabaqabeGadaaakeaacqWGqbaucqGGOaakieqacqWF6bGEcqGG8baFiiGacqGFZoWzcqGGPaqkcqGH9aqpdaWdraqaaiabdcfaqjabcIcaOiab=Pha6jabcYha8jab=rha0jabcMcaPiabdcfaqjabcIcaOiab=rha0jabcYha8jab=HfaynaaBaaaleaacqGFZoWzaeqaaOGaeiykaKIaemizaqMae8hDaqNaeiOFa4NaemOta4KaeiikaGIaemOyaiMae8xmaeZaaSbaaSqaaiab=5gaUbqabaGccqGGSaalcqWFlbWsdaWgaaWcbaGae43SdCgabeaakiabgUcaRGGabiab9L6axjabcMcaPaWcbaGae8hDaqhabeqdcqGHRiI8aaaa@59F2@

**K**_*γ*,*ij *_= *K*(**x**_*γi *_+ **x**_*γj*_), *i*, *j *= 1,2,..., *n*; **Ω **= *σ*^2^**I_n_**, **I**_**n **_and **1**_**n **_are defined in (7).     (12)

The detailed derivation for (12) is provided in Appendix. After inserting the prior given by (10), we have

P(γ|z)∝[det⁡(Kγ+σ2In)]−12×exp⁡{−12[(z−b1n)'(Kγ+σ2In)−1(z−b1n)]}∏j=1pπjγj(1−πj)1−γj     (13)
 MathType@MTEF@5@5@+=feaafiart1ev1aaatCvAUfKttLearuWrP9MDH5MBPbIqV92AaeXatLxBI9gBaebbnrfifHhDYfgasaacH8akY=wiFfYdH8Gipec8Eeeu0xXdbba9frFj0=OqFfea0dXdd9vqai=hGuQ8kuc9pgc9s8qqaq=dirpe0xb9q8qiLsFr0=vr0=vr0dc8meaabaqaciaacaGaaeqabaqabeGadaaakeaafaqaaeGabaaabaGaemiuaaLaeiikaGccceGae83SdCMaeiiFaWhcbeGae4NEaONaeiykaKIaeyyhIuRaei4waSLagiizaqMaeiyzauMaeiiDaqNaeiikaGIae43saS0aaSbaaSqaaGGaciab9n7aNbqabaGccqGHRaWkcqqFdpWCdaahaaWcbeqaaiabikdaYaaakiab+LeajnaaBaaaleaacqGFUbGBaeqaaOGaeiykaKIaeiyxa01aaWbaaSqabeaacqGHsisldaWcaaqaaiabigdaXaqaaiabikdaYaaaaaGccqGHxdaTaeaacaWLjaGagiyzauMaeiiEaGNaeiiCaaNaei4EaSNaeyOeI0YaaSaaaeaacqaIXaqmaeaacqaIYaGmaaGaei4waSLaeiikaGIae4NEaONaeyOeI0IaemOyaiMae4xmaeZaaSbaaSqaaiab+5gaUbqabaGccqGGPaqkcqGGNaWjcqGGOaakcqGFlbWsdaWgaaWcbaGae03SdCgabeaakiabgUcaRiab9n8aZnaaCaaaleqabaGaeGOmaidaaOGae4xsaK0aaSbaaSqaaiab+5gaUbqabaGccqGGPaqkdaahaaWcbeqaaiabgkHiTiabigdaXaaakiabcIcaOiab+Pha6jabgkHiTiabdkgaIjab+fdaXmaaBaaaleaacqGFUbGBaeqaaOGaeiykaKIaeiyxa0LaeiyFa03aaebCaeaacqqFapaCdaqhaaWcbaGaemOAaOgabaGae03SdC2aaSbaaWqaaiabdQgaQbqabaaaaOGaeiikaGIaeGymaeJaeyOeI0Iae0hWda3aaSbaaSqaaiabdQgaQbqabaGccqGGPaqkdaahaaWcbeqaaiabigdaXiabgkHiTiab9n7aNnaaBaaameaacqWGQbGAaeqaaaaaaSqaaiabdQgaQjabg2da9iabigdaXaqaaiabdchaWbqdcqGHpis1aaaakiaaxMaacaWLjaWaaeWaaeaacqaIXaqmcqaIZaWmaiaawIcacaGLPaaaaaa@95C0@

In practice, rather than sampling **γ **as a vector, we sample it component-wise from

P(γj|z)∝[det⁡(Kγ+σ2In)]−12×exp⁡{−12[(z−b1n)'(Kγ+σ2In)−1(z−b1n)]}πjγj(1−πj)1−γj, for j=1,2,...,p.     (14)
 MathType@MTEF@5@5@+=feaafiart1ev1aaatCvAUfKttLearuWrP9MDH5MBPbIqV92AaeXatLxBI9gBaebbnrfifHhDYfgasaacH8akY=wiFfYdH8Gipec8Eeeu0xXdbba9frFj0=OqFfea0dXdd9vqai=hGuQ8kuc9pgc9s8qqaq=dirpe0xb9q8qiLsFr0=vr0=vr0dc8meaabaqaciaacaGaaeqabaqabeGadaaakeaafaqaaeGabaaabaGaemiuaaLaeiikaGccceGae83SdC2aaSbaaSqaaGqaciab+PgaQbqabaGccqGG8baFieqacqqF6bGEcqGGPaqkcqGHDisTcqGGBbWwcyGGKbazcqGGLbqzcqGG0baDcqGGOaakcqqFlbWsdaWgaaWcbaacciGaeW3SdCgabeaakiabgUcaRiab8n8aZnaaCaaaleqabaGaeGOmaidaaOGae0xsaK0aaSbaaSqaaiab95gaUbqabaGccqGGPaqkcqGGDbqxdaahaaWcbeqaaiabgkHiTmaalaaabaGaeGymaedabaGaeGOmaidaaaaakiabgEna0cqaaiaaxMaacyGGLbqzcqGG4baEcqGGWbaCcqGG7bWEcqGHsisldaWcaaqaaiabigdaXaqaaiabikdaYaaacqGGBbWwcqGGOaakcqqF6bGEcqGHsislcqWGIbGycqqFXaqmdaWgaaWcbaGae0NBa4gabeaakiabcMcaPiabcEcaNiabcIcaOiab9TealnaaBaaaleaacqaFZoWzaeqaaOGaey4kaSIaeW3Wdm3aaWbaaSqabeaacqaIYaGmaaGccqqFjbqsdaWgaaWcbaGae0NBa4gabeaakiabcMcaPmaaCaaaleqabaGaeyOeI0IaeGymaedaaOGaeiikaGIae0NEaONaeyOeI0IaemOyaiMae0xmaeZaaSbaaSqaaiab95gaUbqabaGccqGGPaqkcqGGDbqxcqGG9bqFcqaFapaCdaqhaaWcbaGaemOAaOgabaGaeW3SdC2aaSbaaWqaaiabdQgaQbqabaaaaOGaeiikaGIaeGymaeJaeyOeI0IaeWhWda3aaSbaaSqaaiabdQgaQbqabaGccqGGPaqkdaahaaWcbeqaaiabigdaXiabgkHiTiab8n7aNnaaBaaameaacqWGQbGAaeqaaaaaaaGccqGGSaalcqqGGaaicqqGMbGzcqqGVbWBcqqGYbGCcqqGGaaicqWGQbGAcqGH9aqpcqaIXaqmcqGGSaalcqaIYaGmcqGGSaalcqGGUaGlcqGGUaGlcqGGUaGlcqGGSaalcqWGWbaCcqGGUaGlcaWLjaGaaCzcamaabmaabaGaeGymaeJaeGinaqdacaGLOaGaayzkaaaaaa@A2C8@

In both (13) and (14), **K**_*γ *_is defined in (12).

**(II) Sampling from t**|**γ**, *b*, **z**, *σ*^2^, **θ**

As shown by Eq. (A6) in the Appendix, the conditional distribution *P*(**t**|**z**, *b*) is Gaussian:

**t**|**z**, *b *~ *N*((**I**_**n **_- **Ω**(**Ω **+ **K**_*γ*_)^-1^)(**z **- *b***l_n_**), **Ω **- **Ω**(**Ω **+ **K**_**γ**_)^-1^**Ω**),

where **K**_**γ **_and **Ω **are defined in Eq. (12).     (15)

We thus can draw **t **given **z **accordingly.

**(III) Sampling from z**|**t**, *b*, *σ*^2^, **y**

Given the class label vector **y**, the conditional distribution of **z **given **t **is a truncated Gaussian distribution, and we have the following formula for *i *= 1,2,..., *n*:

*z*_*i*_|*t*_*i*_, *b*, *σ*^2^, *y*_*i *_= 1 ∝ *N*(*t*_*i *_+ *b*, *σ*^2^) truncated at the left by 0,

*z*_*i*_|*t*_*i*_, *b*, *σ*^2^, *y*_*i *_= -1 ∝ *N*(*t*_*i *_+ *b*, *σ*^2^) truncated at the right by 0.     (16)

**(IV) Sampling from ***b*|**z**, **t**, *σ*^2^

When **z **and **t **are both given, this is a simple ordinary linear regression setting with only an intercept term. Under the non-informative prior assumption given by (11a), it yields

*b*|**z**, **t**, *σ*^2 ^~ *N*(*μ*, *σ*^2^/*n*), where μ=1n∑i=1n(zi−ti)
 MathType@MTEF@5@5@+=feaafiart1ev1aaatCvAUfKttLearuWrP9MDH5MBPbIqV92AaeXatLxBI9gBaebbnrfifHhDYfgasaacH8akY=wiFfYdH8Gipec8Eeeu0xXdbba9frFj0=OqFfea0dXdd9vqai=hGuQ8kuc9pgc9s8qqaq=dirpe0xb9q8qiLsFr0=vr0=vr0dc8meaabaqaciaacaGaaeqabaqabeGadaaakeaaiiGacqWF8oqBcqGH9aqpdaWcaaqaaiabigdaXaqaaiabd6gaUbaadaaeWbqaaiabcIcaOiabdQha6naaBaaaleaacqWGPbqAaeqaaOGaeyOeI0IaemiDaq3aaSbaaSqaaiabdMgaPbqabaGccqGGPaqkaSqaaiabdMgaPjabg2da9iabigdaXaqaaiabd6gaUbqdcqGHris5aaaa@417B@.     (17a)

**(V) Sampling from ***σ*^2^|**z**, **t**, *b*

With *IG*(*α*, *β*), *α *> 0, *β *> 0, as the prior, the conditional posterior distribution for *σ*^2 ^is also an inverted gamma distribution. That is

*σ*^2^|**z**, **t**, *b *~ *IG*(*α *+ *n*/2, *β *+ *ns*^2^/2), where s2=1n∑i=1n(zi−ti−b)2
 MathType@MTEF@5@5@+=feaafiart1ev1aaatCvAUfKttLearuWrP9MDH5MBPbIqV92AaeXatLxBI9gBaebbnrfifHhDYfgasaacH8akY=wiFfYdH8Gipec8Eeeu0xXdbba9frFj0=OqFfea0dXdd9vqai=hGuQ8kuc9pgc9s8qqaq=dirpe0xb9q8qiLsFr0=vr0=vr0dc8meaabaqaciaacaGaaeqabaqabeGadaaakeaacqWGZbWCdaahaaWcbeqaaiabikdaYaaakiabg2da9maalaaabaGaeGymaedabaGaemOBa4gaamaaqahabaGaeiikaGIaemOEaO3aaSbaaSqaaiabdMgaPbqabaGccqGHsislcqWG0baDdaWgaaWcbaGaemyAaKgabeaakiabgkHiTiabdkgaIjabcMcaPmaaCaaaleqabaGaeGOmaidaaaqaaiabdMgaPjabg2da9iabigdaXaqaaiabd6gaUbqdcqGHris5aaaa@45A4@.     (17b)

#### Kernel parameters tuning

One of the major advantages of kernel-induced learning methods is that one can explore the non-linearity feature of the underlying model for a given learning problem by applying different kernels. It is therefore necessary to discuss the issue of kernel parameter tuning. With the KIGP framework constructed above, this turns out to be rather straightforward.

As in the last section, we denote the kernel parameter(s) as **θ**, which can be either a scalar (e.g. the width parameter of an GK or the degree parameter of an PK) or a vector. For algorithmic convenience, we work with the logarithm of the conditional likelihood for the parameter **θ**:

L(θ)=log⁡(P(z|γ,b,σ2,θ))=−12log⁡(det⁡(Kγ(θ)+σ2In))−12[(z−b1n)'(Kγ(θ)+σ2In)−1(z−b1n)]−n2log⁡(2π)     (18)
 MathType@MTEF@5@5@+=feaafiart1ev1aaatCvAUfKttLearuWrP9MDH5MBPbIqV92AaeXatLxBI9gBaebbnrfifHhDYfgasaacH8akY=wiFfYdH8Gipec8Eeeu0xXdbba9frFj0=OqFfea0dXdd9vqai=hGuQ8kuc9pgc9s8qqaq=dirpe0xb9q8qiLsFr0=vr0=vr0dc8meaabaqaciaacaGaaeqabaqabeGadaaakeaafaqabeGabaaabaGaemitaWKaeiikaGccceGae8hUdeNaeiykaKIaeyypa0JagiiBaWMaei4Ba8Maei4zaCMaeiikaGIaemiuaaLaeiikaGccbeGae4NEaONaeiiFaWNae83SdCMaeiilaWIaemOyaiMaeiilaWccciGae03Wdm3aaWbaaSqabeaacqaIYaGmaaGccqGGSaalcqWF4oqCcqGGPaqkcqGGPaqkcqGH9aqpcqGHsisldaWcaaqaaiabigdaXaqaaiabikdaYaaacyGGSbaBcqGGVbWBcqGGNbWzcqGGOaakcyGGKbazcqGGLbqzcqGG0baDcqGGOaakcqGFlbWsdaWgaaWcbaGae03SdCgabeaakiabcIcaOiab=H7aXjabcMcaPiabgUcaRiab9n8aZnaaCaaaleqabaGaeGOmaidaaOGae4xsaK0aaSbaaSqaaiab+5gaUbqabaGccqGGPaqkcqGGPaqkcqGHsislaeaadaWcaaqaaiabigdaXaqaaiabikdaYaaacqGGBbWwcqGGOaakcqGF6bGEcqGHsislcqWGIbGycqGFXaqmdaWgaaWcbaGae4NBa4gabeaakiabcMcaPiabcEcaNiabcIcaOiab+TealnaaBaaaleaacqqFZoWzaeqaaOGaeiikaGIae8hUdeNaeiykaKIaey4kaSIae03Wdm3aaWbaaSqabeaacqaIYaGmaaGccqGFjbqsdaWgaaWcbaGae4NBa4gabeaakiabcMcaPmaaCaaaleqabaGaeyOeI0IaeGymaedaaOGaeiikaGIae4NEaONaeyOeI0IaemOyaiMae4xmaeZaaSbaaSqaaiab+5gaUbqabaGccqGGPaqkcqGGDbqxcqGHsisldaWcaaqaaiabd6gaUbqaaiabikdaYaaacyGGSbaBcqGGVbWBcqGGNbWzcqGGOaakcqaIYaGmcqqFapaCcqGGPaqkaaGaaCzcaiaaxMaadaqadaqaaiabigdaXiabiIda4aGaayjkaiaawMcaaaaa@9B75@

With a proper prior distribution for **θ**, *P*(**θ**), we have:

*P*(**θ**|**z**, **γ**, *b*, *σ*^2^) ∝ exp (*L*(**θ**))* *P*(**θ**),     (19)

where *L*(**θ**) is defined in (18). In this paper, we specifically focus on two kernel types: the polynomial kernel and the Gaussian kernel, as defined in (6b) and (6c) respectively. For an GK, with the prior for the width parameter given in the "Prior specification" subsection, the resulted posterior distribution given by (19) is non-regular. We apply the Metropolis-Hasting algorithm (the details can be found in [31]) to draw the sample. For an PK, we simply calculate the likelihood with respect to each *d *by (18) and sample *d *accordingly. Sometimes, one may need to calculate the gradient of *L*(**θ**) with respect to **θ **(assume **θ **= [*θ*_1_,..., *θ*_*J*_]') when adopting other plausible algorithms:

∂L(θ)∂θi=−12Trace[Ωγ(θ)−1∂Ωγ(θ)∂θi]+12[(z−b1n)'Ωγ(θ)−1∂Ωγ(θ)∂θiΩγ(θ)−1(z−b1n)],
 MathType@MTEF@5@5@+=feaafiart1ev1aaatCvAUfKttLearuWrP9MDH5MBPbIqV92AaeXatLxBI9gBaebbnrfifHhDYfgasaacH8akY=wiFfYdH8Gipec8Eeeu0xXdbba9frFj0=OqFfea0dXdd9vqai=hGuQ8kuc9pgc9s8qqaq=dirpe0xb9q8qiLsFr0=vr0=vr0dc8meaabaqaciaacaGaaeqabaqabeGadaaakeaadaWcaaqaaiabgkGi2kabdYeamjabcIcaOGGabiab=H7aXjabcMcaPaqaaiabgkGi2IGaciab+H7aXnaaBaaaleaacqWGPbqAaeqaaaaakiabg2da9iabgkHiTmaalaaabaGaeGymaedabaGaeGOmaidaaiabdsfaujabdkhaYjabdggaHjabdogaJjabdwgaLjabcUfaBjabfM6axnaaBaaaleaacqGFZoWzaeqaaOGaeiikaGIae8hUdeNaeiykaKYaaWbaaSqabeaacqGHsislcqaIXaqmaaGcdaWcaaqaaiabgkGi2kabfM6axnaaBaaaleaacqGFZoWzaeqaaOGaeiikaGIae8hUdeNaeiykaKcabaGaeyOaIyRae4hUde3aaSbaaSqaaiabdMgaPbqabaaaaOGaeiyxa0Laey4kaSYaaSaaaeaacqaIXaqmaeaacqaIYaGmaaGaei4waSLaeiikaGccbeGae0NEaONaeyOeI0IaemOyaiMae0xmaeZaaSbaaSqaaiab95gaUbqabaGccqGGPaqkcqGGNaWjcqqHPoWvdaWgaaWcbaGae43SdCgabeaakiabcIcaOiab=H7aXjabcMcaPmaaCaaaleqabaGaeyOeI0IaeGymaedaaOWaaSaaaeaacqGHciITcqqHPoWvdaWgaaWcbaGae43SdCgabeaakiabcIcaOiab=H7aXjabcMcaPaqaaiabgkGi2kab+H7aXnaaBaaaleaacqWGPbqAaeqaaaaakiabfM6axnaaBaaaleaacqGFZoWzaeqaaOGaeiikaGIae8hUdeNaeiykaKYaaWbaaSqabeaacqGHsislcqaIXaqmaaGccqGGOaakcqqF6bGEcqGHsislcqWGIbGycqqFXaqmdaWgaaWcbaGae0NBa4gabeaakiabcMcaPiabc2faDjabcYcaSaaa@8F80@

where Ω_*γ*_(**θ**) = **K**_*γ *_(**θ**) + *σ*^2^**I_n_**, *i *= 1,..., *J*.     (20)

Theoretically, the proposed KIGP with the linear kernel performs very close to most other classical linear methods. As the width parameter of a Gaussian kernel increases (bigger and bigger than 1), within a reasonable range, the KIGP with such an GK performs fairly close to the KIGP with a linear kernel. On the contrary, when the width decreases (smaller and smaller than 1), the performance of the KIGP in the observation space behaves very non-linear. When the degree of a polynomial kernel of an KIGP increases, the non-linearity of the KIGP also increases. When the degree is equal to 1, the only difference between the PK(1) and the linear kernel is a constant. In short, within a kernel class, different values of the kernel parameter represent different feature spaces. For certain specific kernel parameter values, the performance of the KIGP with an GK or with an PK will be close to the KIGP with a linear kernel or a classical linear model in general. Therefore, the posterior distribution of the kernel parameter will provide some clues on what kind of a feature space is more appropriate to the target problem with the given training samples.

#### Proposed Gibbs sampler

With the derivation above and a given kernel type, we propose our Gibbs sampling algorithm as follows:

1. Start with proper initial value [**γ**^[0]^, *b*^[0]^, **t**^[0]^, **z**^[0]^, *σ*^2[0]^, *θ*^[0]^]; then set *i *= 1.

2. Sample **z **[*i*] from **z**|**t**^[*i*-1]^, *b*^[*i*-1]^, *σ*^2[*i*-1] ^via (16).

3. Sample **t**^[*i*] ^from **t**|**γ**^[*i*-1]^, *b*^[*i*-1]^, **z**^[*i*]^, *σ*^2[*i*-1]^, *θ*^[*i*-1] ^via (15).

4. Sample *b*^[*i*] ^from *b*|**z**^[*i*]^, **t**^[*i*]^, *σ*^2[*i*-1] ^via (17a).

5. Sample *σ*^2[*i*] ^from *σ*^2^|**z**^[*i*]^, **t**^[*i*]^, *b*^[*i*] ^via (17b).

6. Sample **γ**^[*i*] ^from **γ**|**z**^[*i*]^, *b*^[*i*]^, *σ*^2[*i*]^, *θ*^[*i*-1] ^via (14) component-wise.

7. Sample *θ*^[*i*] ^from *θ*|**z**^[*i*]^, *b*^[*i*]^, *σ*^2[*i*]^, **γ**^[*i*]^.

8. Set *i *= *i *+ 1 and go back to the step 2 until the required number of iterations.

9. Stop.     (21)

In the above procedure, the kernel parameter *θ *denotes the degree parameter "d" of a polynomial kernel or the width parameter "r" of a Gaussian kernel. In step 2, we follow the optimal exponential accept-reject algorithm suggested by Robert [32] to draw from a truncated Gaussian distribution. After a suitable burn-in period, we can obtain the posterior samples of [**z**^[*i*]^, **t**^[*i*]^, *b*^[*i*]^, *σ*^2[*i*]^, **γ**^[*i*]^, *θ*^[*i*]^] at the *i *th iteration with the procedure described in (21). The core calculation of the proposed Gibbs sampler involves calculating the inverse of the matrix **K**_*γ *_+ *σ*^2^**I**. Since the kernel matrix **K**_*γ *_is symmetric and non-negative definite, **K**_*γ *_+ *σ*^2^**I **is symmetric and positive definite. Therefore, the algorithm is theoretically robust and the Cholesky decomposition can be applied in the numerical computation. The total computation complexity of the proposed Gibbs sampler within each iteration is *O*(*pn*^3^).

#### Overall algorithm

In Fig. 1, we epitomize the general framework of the proposed KIGP method. The box bounded by the dotted lines represents the KIGP learning algorithm. A kernel type is supposed to be given a priori. The algorithm basically has a cascading structure and is composed of three consecutive phases: the "kernel parameter fitting phase", the "gene selection phase" and the "prediction phase". Although in the Bayesian sense one can involve all the parameters into the proposed Gibbs sampler for all three phases, we suggest to fix the kernel parameter(s) after the "kernel parameter fitting phase" and fix the gene-selection vector after the "gene selection phase" for practicality. Very often, we are only interested in the area around the peak of the posterior PDF (or probability mass function (PMF)) of a parameter, especially for the kernel parameter(s) and the gene-selection vector. This strategy will lead to a much faster convergence of the proposed Gibbs sampler as long as the posterior PDF or PMF of the kernel parameter(s) is unimodal. For all three phases, we need to discard some proper number of iterations as their burn-in periods. Some dynamic monitoring strategies to track the convergence of a MCMC simulation can be used (e.g. in [31]).

A practical issue needs to be addressed here. It's better to fix the variance parameter *σ*^2 ^at a proper constant during the "kernel parameter fitting phase" and the "gene selection phase" because this will help the proposed algorithm be more numerically stable and converge faster. For all the simulations of this paper, as in a regular probit regression model, we set *σ*^2 ^equal to 1 (step 5 in (21)) in the first two phases and only involve it into the Gibbs sampler in the "prediction phase". More details of each phase are described as follows.

#### Kernel Parameter Fitting Phase

In the kernel parameter fitting phase, our primary interest is to find the appropriate value(s) for the kernel parameter(s) of the given kernel type. In this study, we focus on two kernel types, the polynomial kernel and the Gaussian kernel. With the knowledge of the training set **X **and **y**, we firstly involve all model parameters (except *σ*^2^), the gene selection vector and the kernel parameter into the simulation of the algorithm given by (21). After convergence, the samples obtained from (21) within each iteration are drawn from the joint posterior distribution of all the parameters. For a PK, since the degree parameter is a discrete number, we simply take the degree value with the highest posterior probability. For a GK, we calculate the histogram of the sample values of the width parameter with some proper number of bins. Then we use a Gaussian smoother to smooth over the histogram bars (similar to a Gaussian kernel density estimation). Finally, we take the center of the bin with the highest histogram counts as the best fitted value of the width parameter.

#### Gene Selection Phase

After the "kernel parameter fitting phase", we fix the kernel parameter(s) at the fitted value(s) and then continue to run the proposed Gibbs sampler. In this subsection, we present an empirical approach to determining whether a gene is potentially significant based on the posterior samples and a given threshold.

Efron [29] thoroughly discussed an empirical Bayes approach for estimating the null hypothesis based on a large-scale simultaneous t-test. In this paper, we essentially follow the key concept therein to assess whether or not a gene is of significant importance for the given classification problem. We first define a statistic named by "Normalized Log-Frequency" (NLF) to measure the relative potential significance for a gene. By denoting *F*_*j *_as the appearing frequency of the *j *th gene appeared in the posterior samples, the definition of NLF is formulated as:

NLFj=LFj−μLsL, where LFj=log⁡(Fj)μL=1p∑j=1pLFj, sL2=1p−1∑j=1P(LFj−μL)2  for  j=1,2,...,p.     (22)
 MathType@MTEF@5@5@+=feaafiart1ev1aaatCvAUfKttLearuWrP9MDH5MBPbIqV92AaeXatLxBI9gBaebbnrfifHhDYfgasaacH8akY=wiFfYdH8Gipec8Eeeu0xXdbba9frFj0=OqFfea0dXdd9vqai=hGuQ8kuc9pgc9s8qqaq=dirpe0xb9q8qiLsFr0=vr0=vr0dc8meaabaqaciaacaGaaeqabaqabeGadaaakeaafaqaaeGabaaabaGaemOta4KaemitaWKaemOray0aaSbaaSqaaiabdQgaQbqabaGccqGH9aqpdaWcaaqaaiabdYeamjabdAeagnaaBaaaleaacqWGQbGAaeqaaOGaeyOeI0ccciGae8hVd02aaSbaaSqaaiabdYeambqabaaakeaacqWGZbWCdaWgaaWcbaGaemitaWeabeaaaaGccqGGSaalcqqGGaaicqqG3bWDcqqGObaAcqqGLbqzcqqGYbGCcqqGLbqzcqqGGaaicqWGmbatcqWGgbGrdaWgaaWcbaGaemOAaOgabeaakiabg2da9iGbcYgaSjabc+gaVjabcEgaNjabcIcaOiabdAeagnaaBaaaleaacqWGQbGAaeqaaOGaeiykaKcabaGae8hVd02aaSbaaSqaaiabdYeambqabaGccqGH9aqpdaWcaaqaaiabigdaXaqaaiabdchaWbaadaaeWbqaaiabdYeamjabdAeagnaaBaaaleaacqWGQbGAaeqaaOGaeiilaWIaeeiiaacaleaacqWGQbGAcqGH9aqpcqaIXaqmaeaacqWGWbaCa0GaeyyeIuoakiabdohaZnaaDaaaleaacqWGmbataeaacqaIYaGmaaGccqGH9aqpdaWcaaqaaiabigdaXaqaaiabdchaWjabgkHiTiabigdaXaaadaaeWbqaaiabcIcaOiabdYeamjabdAeagnaaBaaaleaacqWGQbGAaeqaaOGaeyOeI0Iae8hVd02aaSbaaSqaaiabdYeambqabaGccqGGPaqkdaahaaWcbeqaaiabikdaYaaakiabbccaGiabbccaGiabbAgaMjabb+gaVjabbkhaYjabbccaGiabbccaGiabdQgaQjabg2da9iabigdaXiabcYcaSiabikdaYiabcYcaSiabc6caUiabc6caUiabc6caUiabcYcaSiabdchaWbWcbaGaemOAaOMaeyypa0JaeGymaedabaGaemiuaafaniabggHiLdGccqGGUaGlaaGaaCzcaiaaxMaadaqadaqaaiabikdaYiabikdaYaGaayjkaiaawMcaaaaa@98FD@

In practice, if *F*_*j *_is 0, we simply set it as 1/2 divided by the total number of iterations. Our use of the NLF as the key statistic is based on the fact that the logarithm of a gamma distribution can be well approximated by a normal distribution, while a gamma distribution is empirically a proper distribution for the appearing frequency of any of the genes from a homogenous group in the posterior samples.

Suppose that the *p *NLF-values fall into two classes, "insignificant" or "significant", corresponding to whether or not *NLF*_*j*_, for *j *= 1, 2,..., *p*, is generated according to the null hypothesis, with prior probabilities *Pb*_0 _and *Pb*_1 _= 1 - *Pb*_0_, for the two classes respectively; and that *NLF*_*j *_has the conditional prior density either *f*_0 _(*NLF*) or *f*_1_(*NLF*) depending on its class. I.e.

*Pb*_0 _= Pr{*Insignificant*}, Pr(*NLF*|*Insignificant*) = *f*_0_(*NLF*)

*Pb*_1 _= Pr{*Significant*}, Pr(*NLF*|*Significant*) = *f*_1_(*NLF*)     (23)

The marginal distribution for *NLF*_*j *_is thus

Pr(*NLF*) = *f*_0_(*NLF*)*Pb_0 _+ *f*_1_(*NLF*) * *Pb*_1 _= *f *(*NLF*)     (24)

By using the Bayes' formula, the posterior probability for "insignificant" class given the NLF therefore yields

Pr(*Insignificant*)|*NLF*) = *f*_0_(*NLF*) * *Pb*_0_/*f*(*NLF*)     (25)

Abiding to [29], we further define a term, the local "false discovery rate (fdr)", by

*fdr*(*NLF*) = *f*_0_(*NLF*)/*f*(*NLF*)     (26)

Since in a typical microarray study, *Pb*_0 _generally is very close to 1 (say *Pb*_0 _> 0.99), so *fdr*(*NLF*) is a fairly precise estimator for the posterior probability of the null hypothesis (insignificant class) given the statistic NLF. With *fdr*(*NLF*), we can decide whether or not a target gene is "significant" at some confidence level accordingly. For all the examples, we report all the genes with fdr smaller than 0.05.

To calculate *fdr*(*NLF*), one needs to estimate *f*(*NLF*) and to choose *f*_0_(*NLF*) properly. For estimating *f*(*NLF*), one can resort to the ensemble values of the NLFs, {*NLF*_*j*_, *j *= 1, 2,..., *p*}. We divide the target range of NLF into *M *equal length bins with the center of each bin at *x*_*i *_for *i *= 1,2,..., *M*. A heuristic choice of M is the roundup of the maximum NLF value multiplied by 10. Then we calculate the histogram for the given NLF set with respect to each of these bins followed by fitting a Gaussian smoother. The output divided by the product of the width of the bin and the number of genes (i.e. *p*) will be a proper estimation for *f*(*NLF*) on the center of each bin.

The more critical part is the choice of the density of NLF under null hypothesis, i.e. *f*_0_(*NLF*). The basic assumption we impose here is that the statistic NLF under null hypothesis follows a normal distribution. Since *Pb*_1 _is much smaller than *Pb*_0 _(say *Pb*_0 _> 0.99) in most real microarray analysis problems, it is very safe to choose the standard normal (zero mean, unit variance) as *f*_0_(*NLF*) based on the definition (22). Throughout this paper, we always choose the standard normal as the density of NLF under null hypothesis. (In case *Pb*_0 _> 0.99, some more elaborated schemes are needed and an easy approach can be found in [29].) After both *f*(*NLF*) and *f*_0_(*NLF*) are obtained, the local fdr for each gene can be calculated by (26) consequently. Based on the local fdr, one can select the "significant" class of genes and fix the gene-selection vector at some given confidence level thereafter.

#### Prediction Phase

After the "gene selection phase", both the kernel parameter(s) and the gene-selection vector have been fixed. We continue to run the proposed Gibbs sampler (21) and the computational complexity of the Gibbs sampler dramatically decreases to *O*(*n*^3^). After a new proper burn-in period, we can draw samples of **z**, *b *and *σ*^2 ^within each iteration in the "prediction phase". Following (9), the posterior PDF for the output t˜
 MathType@MTEF@5@5@+=feaafiart1ev1aaatCvAUfKttLearuWrP9MDH5MBPbIqV92AaeXatLxBI9gBaebbnrfifHhDYfgasaacH8akY=wiFfYdH8Gipec8Eeeu0xXdbba9frFj0=OqFfea0dXdd9vqai=hGuQ8kuc9pgc9s8qqaq=dirpe0xb9q8qiLsFr0=vr0=vr0dc8meaabaqaciaacaGaaeqabaqabeGadaaakeaacuWG0baDgaacaaaa@2E2C@ given the testing data x˜
 MathType@MTEF@5@5@+=feaafiart1ev1aaatCvAUfKttLearuWrP9MDH5MBPbIqV92AaeXatLxBI9gBaebbnrfifHhDYfgasaacH8akY=wiFfYdH8Gipec8Eeeu0xXdbba9frFj0=OqFfea0dXdd9vqai=hGuQ8kuc9pgc9s8qqaq=dirpe0xb9q8qiLsFr0=vr0=vr0dc8meaabaqaciaacaGaaeqabaqabeGadaaakeaaieqacuWF4baEgaacaaaa@2E3A@ in the *l*-th iteration is Gaussian:

t˜
 MathType@MTEF@5@5@+=feaafiart1ev1aaatCvAUfKttLearuWrP9MDH5MBPbIqV92AaeXatLxBI9gBaebbnrfifHhDYfgasaacH8akY=wiFfYdH8Gipec8Eeeu0xXdbba9frFj0=OqFfea0dXdd9vqai=hGuQ8kuc9pgc9s8qqaq=dirpe0xb9q8qiLsFr0=vr0=vr0dc8meaabaqaciaacaGaaeqabaqabeGadaaakeaacuWG0baDgaacaaaa@2E2C@^[*l*]^|x˜
 MathType@MTEF@5@5@+=feaafiart1ev1aaatCvAUfKttLearuWrP9MDH5MBPbIqV92AaeXatLxBI9gBaebbnrfifHhDYfgasaacH8akY=wiFfYdH8Gipec8Eeeu0xXdbba9frFj0=OqFfea0dXdd9vqai=hGuQ8kuc9pgc9s8qqaq=dirpe0xb9q8qiLsFr0=vr0=vr0dc8meaabaqaciaacaGaaeqabaqabeGadaaakeaaieqacuWF4baEgaacaaaa@2E3A@_*γ*_, **X**_*γ*_, **z**^[*l*]^, *b*^[*l*]^, *σ*^2[*l*] ^~ *N*(*f*(x˜
 MathType@MTEF@5@5@+=feaafiart1ev1aaatCvAUfKttLearuWrP9MDH5MBPbIqV92AaeXatLxBI9gBaebbnrfifHhDYfgasaacH8akY=wiFfYdH8Gipec8Eeeu0xXdbba9frFj0=OqFfea0dXdd9vqai=hGuQ8kuc9pgc9s8qqaq=dirpe0xb9q8qiLsFr0=vr0=vr0dc8meaabaqaciaacaGaaeqabaqabeGadaaakeaaieqacuWF4baEgaacaaaa@2E3A@_*γ*_, **X**_*γ*_, **z**^[*l*]^, *b*^[*l*]^, *σ*^2[*l*]^), *V*(x˜
 MathType@MTEF@5@5@+=feaafiart1ev1aaatCvAUfKttLearuWrP9MDH5MBPbIqV92AaeXatLxBI9gBaebbnrfifHhDYfgasaacH8akY=wiFfYdH8Gipec8Eeeu0xXdbba9frFj0=OqFfea0dXdd9vqai=hGuQ8kuc9pgc9s8qqaq=dirpe0xb9q8qiLsFr0=vr0=vr0dc8meaabaqaciaacaGaaeqabaqabeGadaaakeaaieqacuWF4baEgaacaaaa@2E3A@_*γ*_, **X**_*γ*_, **z**^[*l*]^, *b*^[*l*]^, *σ*^2[*l*]^)) = *N*(*f*^[*l*]^, *V*^[*l*]^)

where *f*^[*l*] ^= (**z**^[*l*] ^- *b*^[*l*]^**l**_**n**_)' (**K**_*γ *_+ *σ*^2[*l*]^**I**_*n*_)^-1^**k**_*γ*_, *V*^[*l*] ^= *K*(x˜
 MathType@MTEF@5@5@+=feaafiart1ev1aaatCvAUfKttLearuWrP9MDH5MBPbIqV92AaeXatLxBI9gBaebbnrfifHhDYfgasaacH8akY=wiFfYdH8Gipec8Eeeu0xXdbba9frFj0=OqFfea0dXdd9vqai=hGuQ8kuc9pgc9s8qqaq=dirpe0xb9q8qiLsFr0=vr0=vr0dc8meaabaqaciaacaGaaeqabaqabeGadaaakeaaieqacuWF4baEgaacaaaa@2E3A@_*γ*_, x˜
 MathType@MTEF@5@5@+=feaafiart1ev1aaatCvAUfKttLearuWrP9MDH5MBPbIqV92AaeXatLxBI9gBaebbnrfifHhDYfgasaacH8akY=wiFfYdH8Gipec8Eeeu0xXdbba9frFj0=OqFfea0dXdd9vqai=hGuQ8kuc9pgc9s8qqaq=dirpe0xb9q8qiLsFr0=vr0=vr0dc8meaabaqaciaacaGaaeqabaqabeGadaaakeaaieqacuWF4baEgaacaaaa@2E3A@_*γ*_) - **k**_*γ*_'(**K**_*γ *_+ *σ*^2[*l*]^**I**_*n*_)^-1^**k**_*γ*_,

**K**_*γ,ij*_, = *K*(**x**_*γ*,**i**_, **x**_*γ*,**j**_), **k**_*γ,i *_= *K*(x˜
 MathType@MTEF@5@5@+=feaafiart1ev1aaatCvAUfKttLearuWrP9MDH5MBPbIqV92AaeXatLxBI9gBaebbnrfifHhDYfgasaacH8akY=wiFfYdH8Gipec8Eeeu0xXdbba9frFj0=OqFfea0dXdd9vqai=hGuQ8kuc9pgc9s8qqaq=dirpe0xb9q8qiLsFr0=vr0=vr0dc8meaabaqaciaacaGaaeqabaqabeGadaaakeaaieqacuWF4baEgaacaaaa@2E3A@_*γ*_, **x**_*γ,i*_), for *i*, *j *= 1,..., *n *; *l *= 1,..., *L*.     (27a)

Then, the predictive probability for the output label y˜
 MathType@MTEF@5@5@+=feaafiart1ev1aaatCvAUfKttLearuWrP9MDH5MBPbIqV92AaeXatLxBI9gBaebbnrfifHhDYfgasaacH8akY=wiFfYdH8Gipec8Eeeu0xXdbba9frFj0=OqFfea0dXdd9vqai=hGuQ8kuc9pgc9s8qqaq=dirpe0xb9q8qiLsFr0=vr0=vr0dc8meaabaqaciaacaGaaeqabaqabeGadaaakeaacuWG5bqEgaacaaaa@2E36@ given x˜
 MathType@MTEF@5@5@+=feaafiart1ev1aaatCvAUfKttLearuWrP9MDH5MBPbIqV92AaeXatLxBI9gBaebbnrfifHhDYfgasaacH8akY=wiFfYdH8Gipec8Eeeu0xXdbba9frFj0=OqFfea0dXdd9vqai=hGuQ8kuc9pgc9s8qqaq=dirpe0xb9q8qiLsFr0=vr0=vr0dc8meaabaqaciaacaGaaeqabaqabeGadaaakeaaieqacuWF4baEgaacaaaa@2E3A@ can be estimated by using the Monte Carlo integration:

P(y˜=1|X,y,x˜)=1L∑l=1LΦ(f[l]+b[l]V[l]),P(y˜=−1|X,y,x˜)=1−P(y˜=1|X,y,x˜),where     Φ(x)=∫−∞x12πe−t2/2dt.     (27b)
 MathType@MTEF@5@5@+=feaafiart1ev1aaatCvAUfKttLearuWrP9MDH5MBPbIqV92AaeXatLxBI9gBaebbnrfifHhDYfgasaacH8akY=wiFfYdH8Gipec8Eeeu0xXdbba9frFj0=OqFfea0dXdd9vqai=hGuQ8kuc9pgc9s8qqaq=dirpe0xb9q8qiLsFr0=vr0=vr0dc8meaabaqaciaacaGaaeqabaqabeGadaaakeaafaqaaeGabaaabaGaemiuaaLaeiikaGIafmyEaKNbaGaacqGH9aqpcqaIXaqmcqGG8baFieqacqWFybawcqGGSaalcqWF5bqEcqGGSaalcuWF4baEgaacaiabcMcaPiabg2da9maalaaabaGaeGymaedabaGaemitaWeaamaaqahabaGaeuOPdyealeaacqWGSbaBcqGH9aqpcqaIXaqmaeaacqWGmbata0GaeyyeIuoakiabcIcaOmaalaaabaGaemOzay2aaWbaaSqabeaacqGGBbWwcqWGSbaBcqGGDbqxaaGccqGHRaWkcqWGIbGydaahaaWcbeqaaiabcUfaBjabdYgaSjabc2faDbaaaOqaamaakaaabaGaemOvay1aaWbaaSqabeaacqGGBbWwcqWGSbaBcqGGDbqxaaaabeaaaaGccqGGPaqkcqGGSaalcqWGqbaucqGGOaakcuWG5bqEgaacaiabg2da9iabgkHiTiabigdaXiabcYha8jab=HfayjabcYcaSiab=Lha5jabcYcaSiqb=Hha4zaaiaGaeiykaKIaeyypa0JaeGymaeJaeyOeI0IaemiuaaLaeiikaGIafmyEaKNbaGaacqGH9aqpcqaIXaqmcqGG8baFcqWFybawcqGGSaalcqWF5bqEcqGGSaalcuWF4baEgaacaiabcMcaPiabcYcaSaqaaiabbEha3jabbIgaOjabbwgaLjabbkhaYjabbwgaLjabbccaGiabbccaGiabbccaGiabbccaGiabbccaGiabfA6agjabcIcaOiabdIha4jabcMcaPiabg2da9maapedabaWaaSaaaeaacqaIXaqmaeaadaGcaaqaaiabikdaYGGaciab+b8aWbWcbeaaaaGccqWGLbqzdaahaaWcbeqaaiabgkHiTiabdsha0naaCaaameqabaGaeGOmaidaaSGaei4la8IaeGOmaidaaOGaemizaqMaemiDaqNaeiOla4caleaacqGHsislcqGHEisPaeaacqWG4baEa0Gaey4kIipaaaGccaWLjaGaaCzcamaabmaabaGaeGOmaiJaeG4naCJaeeOyaigacaGLOaGaayzkaaaaaa@A4CD@

#### Kernel type competition

Another important issue needs to be addressed is how to properly select a kernel type. If an independent set of testing samples is available, one approach is to calculate its predictive fit measure such as the misclassification rate (MR) or average predictive probability (APP) of the true class label. If the number of the available testing samples is sufficiently large, this approach is very reliable.

Assuming that there are *M *testing samples {(x˜
 MathType@MTEF@5@5@+=feaafiart1ev1aaatCvAUfKttLearuWrP9MDH5MBPbIqV92AaeXatLxBI9gBaebbnrfifHhDYfgasaacH8akY=wiFfYdH8Gipec8Eeeu0xXdbba9frFj0=OqFfea0dXdd9vqai=hGuQ8kuc9pgc9s8qqaq=dirpe0xb9q8qiLsFr0=vr0=vr0dc8meaabaqaciaacaGaaeqabaqabeGadaaakeaaieqacuWF4baEgaacaaaa@2E3A@_1_, y⌢
 MathType@MTEF@5@5@+=feaafiart1ev1aaatCvAUfKttLearuWrP9MDH5MBPbIqV92AaeXatLxBI9gBaebbnrfifHhDYfgasaacH8akY=wiFfYdH8Gipec8Eeeu0xXdbba9frFj0=OqFfea0dXdd9vqai=hGuQ8kuc9pgc9s8qqaq=dirpe0xb9q8qiLsFr0=vr0=vr0dc8meaabaqaciaacaGaaeqabaqabeGadaaakeaacuWG5bqEgaWeaaaa@2E41@_1_),...,(x˜
 MathType@MTEF@5@5@+=feaafiart1ev1aaatCvAUfKttLearuWrP9MDH5MBPbIqV92AaeXatLxBI9gBaebbnrfifHhDYfgasaacH8akY=wiFfYdH8Gipec8Eeeu0xXdbba9frFj0=OqFfea0dXdd9vqai=hGuQ8kuc9pgc9s8qqaq=dirpe0xb9q8qiLsFr0=vr0=vr0dc8meaabaqaciaacaGaaeqabaqabeGadaaakeaaieqacuWF4baEgaacaaaa@2E3A@_*M*_, y⌢
 MathType@MTEF@5@5@+=feaafiart1ev1aaatCvAUfKttLearuWrP9MDH5MBPbIqV92AaeXatLxBI9gBaebbnrfifHhDYfgasaacH8akY=wiFfYdH8Gipec8Eeeu0xXdbba9frFj0=OqFfea0dXdd9vqai=hGuQ8kuc9pgc9s8qqaq=dirpe0xb9q8qiLsFr0=vr0=vr0dc8meaabaqaciaacaGaaeqabaqabeGadaaakeaacuWG5bqEgaWeaaaa@2E41@_*M*_)}, where x˜
 MathType@MTEF@5@5@+=feaafiart1ev1aaatCvAUfKttLearuWrP9MDH5MBPbIqV92AaeXatLxBI9gBaebbnrfifHhDYfgasaacH8akY=wiFfYdH8Gipec8Eeeu0xXdbba9frFj0=OqFfea0dXdd9vqai=hGuQ8kuc9pgc9s8qqaq=dirpe0xb9q8qiLsFr0=vr0=vr0dc8meaabaqaciaacaGaaeqabaqabeGadaaakeaaieqacuWF4baEgaacaaaa@2E3A@_*i *_denotes the microarray data and y⌢
 MathType@MTEF@5@5@+=feaafiart1ev1aaatCvAUfKttLearuWrP9MDH5MBPbIqV92AaeXatLxBI9gBaebbnrfifHhDYfgasaacH8akY=wiFfYdH8Gipec8Eeeu0xXdbba9frFj0=OqFfea0dXdd9vqai=hGuQ8kuc9pgc9s8qqaq=dirpe0xb9q8qiLsFr0=vr0=vr0dc8meaabaqaciaacaGaaeqabaqabeGadaaakeaacuWG5bqEgaWeaaaa@2E41@_*i *_is its class label for *i *= 1,2,..., *M*, the MR for the testing set can be estimated by

MRtest=1M∑i=1MMCi,  whereMCi={1if y˜i≠y⌢i0if y˜i=y⌢i,y˜i={1if P(y˜i=1|X,y,x˜i,K)|≥0.5−1if P(y˜i=1|X,y,x˜i,K)|<0.5  for  i=1,2,...,M     (28a)
 MathType@MTEF@5@5@+=feaafiart1ev1aaatCvAUfKttLearuWrP9MDH5MBPbIqV92AaeXatLxBI9gBaebbnrfifHhDYfgasaacH8akY=wiFfYdH8Gipec8Eeeu0xXdbba9frFj0=OqFfea0dXdd9vqai=hGuQ8kuc9pgc9s8qqaq=dirpe0xb9q8qiLsFr0=vr0=vr0dc8meaabaqaciaacaGaaeqabaqabeGadaaakeaafaqaaeGabaaabaGaemyta0KaemOuai1aaSbaaSqaaiabdsha0jabdwgaLjabdohaZjabdsha0bqabaGccqGH9aqpdaWcaaqaaiabigdaXaqaaiabd2eanbaadaaeWbqaaiabd2eanjabdoeadnaaBaaaleaacqWGPbqAaeqaaOGaeiilaWcaleaacqWGPbqAcqGH9aqpcqaIXaqmaeaacqWGnbqta0GaeyyeIuoakiabbccaGiabbccaGiabbEha3jabbIgaOjabbwgaLjabbkhaYjabbwgaLbqaaiabd2eanjabdoeadnaaBaaaleaacqWGPbqAaeqaaOGaeyypa0ZaaiqabeaafaqabeGacaaabaGaeGymaedabaacbiGae8xAaKMae8NzayMaeeiiaaIafmyEaKNbaGaadaWgaaWcbaGaemyAaKgabeaakiabgcMi5kqbdMha5zaataWaaSbaaSqaaiabdMgaPbqabaaakeaacqaIWaamaeaacqWFPbqAcqWFMbGzcqqGGaaicuWG5bqEgaacamaaBaaaleaacqWGPbqAaeqaaOGaeyypa0JafmyEaKNbambadaWgaaWcbaGaemyAaKgabeaaaaGccqGGSaalcuWG5bqEgaacamaaBaaaleaacqWGPbqAaeqaaOGaeyypa0ZaaiqabeaafaqabeGacaaabaGaeGymaedabaGae8xAaKMae8NzayMaeeiiaaIaemiuaaLaeiikaGIafmyEaKNbaGaadaWgaaWcbaGaemyAaKgabeaakiabg2da9iabigdaXiabcYha8Hqabiab+HfayjabcYcaSiab+Lha5jabcYcaSiqb+Hha4zaaiaWaaSbaaSqaaiabdMgaPbqabaGccqGGSaalieaacqqFlbWscqGGPaqkcqGG8baFcqGHLjYScqaIWaamcqGGUaGlcqaI1aqnaeaacqGHsislcqaIXaqmaeaacqWFPbqAcqWFMbGzcqqGGaaicqWGqbaucqGGOaakcuWG5bqEgaacamaaBaaaleaacqWGPbqAaeqaaOGaeyypa0JaeGymaeJaeiiFaWNae4hwaGLaeiilaWIae4xEaKNaeiilaWIaf4hEaGNbaGaadaWgaaWcbaGaemyAaKgabeaakiabcYcaSiab9TealjabcMcaPiabcYha8jabgYda8iabicdaWiabc6caUiabiwda1aaaaiaawUhaaaGaay5EaaGaeeiiaaIaeeiiaaIaeeOzayMaee4Ba8MaeeOCaiNaeeiiaaIaeeiiaaIaemyAaKMaeyypa0JaeGymaeJaeiilaWIaeGOmaiJaeiilaWIaeiOla4IaeiOla4IaeiOla4IaeiilaWIaemyta0eaaiaaxMaacaWLjaWaaeWaaeaacqaIYaGmcqaI4aaocqqGHbqyaiaawIcacaGLPaaaaaa@C2E4@

The smaller the MR a kernel type has, the better general performance it should have. If the number of the available testing samples is small, the APP of the true class label is a more consistent measure. Throughout this paper, we always refer APP to the APP of the true class label and it is defined as:

APPtest=1M∑i=1MP(y˜i=y⌢i|X,y,x˜i,K).     (28b)
 MathType@MTEF@5@5@+=feaafiart1ev1aaatCvAUfKttLearuWrP9MDH5MBPbIqV92AaeXatLxBI9gBaebbnrfifHhDYfgasaacH8akY=wiFfYdH8Gipec8Eeeu0xXdbba9frFj0=OqFfea0dXdd9vqai=hGuQ8kuc9pgc9s8qqaq=dirpe0xb9q8qiLsFr0=vr0=vr0dc8meaabaqaciaacaGaaeqabaqabeGadaaakeaacqWGbbqqcqWGqbaucqWGqbaudaWgaaWcbaGaemiDaqNaemyzauMaem4CamNaemiDaqhabeaakiabg2da9maalaaabaGaeGymaedabaGaemyta0eaamaaqahabaGaemiuaaLaeiikaGIafmyEaKNbaGaadaWgaaWcbaGaemyAaKgabeaakiabg2da9iqbdMha5zaataWaaSbaaSqaaiabdMgaPbqabaGccqGG8baFieqacqWFybawcqGGSaalcqWF5bqEcqGGSaalcuWF4baEgaacamaaBaaaleaacqWGPbqAaeqaaOGaeiilaWIaem4saSKaeiykaKIaeiOla4caleaacqWGPbqAcqGH9aqpcqaIXaqmaeaacqWGnbqta0GaeyyeIuoakiaaxMaacaWLjaWaaeWaaeaacqaIYaGmcqaI4aaocqqGIbGyaiaawIcacaGLPaaaaaa@5BE4@

In both (28a) and (28b), the probability *P*(y˜
 MathType@MTEF@5@5@+=feaafiart1ev1aaatCvAUfKttLearuWrP9MDH5MBPbIqV92AaeXatLxBI9gBaebbnrfifHhDYfgasaacH8akY=wiFfYdH8Gipec8Eeeu0xXdbba9frFj0=OqFfea0dXdd9vqai=hGuQ8kuc9pgc9s8qqaq=dirpe0xb9q8qiLsFr0=vr0=vr0dc8meaabaqaciaacaGaaeqabaqabeGadaaakeaacuWG5bqEgaacaaaa@2E36@_*i*_|**X**, **y**, x˜
 MathType@MTEF@5@5@+=feaafiart1ev1aaatCvAUfKttLearuWrP9MDH5MBPbIqV92AaeXatLxBI9gBaebbnrfifHhDYfgasaacH8akY=wiFfYdH8Gipec8Eeeu0xXdbba9frFj0=OqFfea0dXdd9vqai=hGuQ8kuc9pgc9s8qqaq=dirpe0xb9q8qiLsFr0=vr0=vr0dc8meaabaqaciaacaGaaeqabaqabeGadaaakeaaieqacuWF4baEgaacaaaa@2E3A@_*i*_, K) is evaluated by (27a) and (27b). Obviously, a better model should have a higher APP. The APP usually provides a less biased predictive fit measure when the number of testing samples is limited.

After running the simulations under each candidate kernel type, one can simply choose the kernel type with the least MR or with the largest APP for the testing set. However, the independent testing samples are not always available. To use the predictive fit approach, one may resort to a rigorous cross-validation (CV) procedure. Sometimes, a "leave-one-out" cross-validation (LOOCV) is proper. That is, one treats one of the training samples as the testing sample and applies the proposed KIGP, including all three phases, to the rest n-1 samples and obtains the predictive measure for this sample. One does this procedure for each training sample and the average of the predictive measures should give a consistent evaluation to the target kernel type.

A more unbiased approach is to use a multiple independent 3-fold CVs. For each round of CV, one first randomly partitions the training set into three sets with a balanced ratio of the class labels. Then for each of the three sets, one treats it as the testing set and applies the KIGP (including all three phases) to the remaining two sets as the training set and gets the predictive fit measure for this testing set. After running this procedure for all three sets, one gets the predictive measure of all available samples for this round. One does multiple rounds of independent 3-fold CVs (through different random partitioning) and the average of the predictive measure for the whole set will deliver an unbiased assessment of the given kernel type.

The predictive fit approach through a multiple 3-fold CVs works very well. Throughout this study, we always use it to select the proper kernel type for a given problem if the independent testing set is not available. As the nature of the MCMC-based methods however, the KIGP method is extremely computationally intensive, especially when the number of the candidate kernel types is large. A more integrative implementation for kernel or model selection, such as making use of a reversible jump MCMC approach, would help further streamline the current KIGP.

## Abbreviations

KIGP kernel-imbedded Gaussian process

AIC Akaike information criterion

BIC Bayesian information criterion

MCMC Markov chain Monte Carlo

PAM prediction analysis for Microarrays

SVM support vector machine

RFE recursive feature elimination

LSSVM least square support vector machine

GP Gaussian process

LK linear kernel

PK polynomial kernel

PK(d) polynomial kernel with degree "d"

GK Gaussian kernel

GK(r) Gaussian kernel with width "r"

NLF normalized log-frequency

fdr false discovery rate

MR misclassification rate

APP average predictive probability

LOOCV leave-one-out cross-validation

3-fold CV 3-fold cross-validation

PDF probability density function

AML acute myeloid leukemia

ALL acute lymphoblastic leukemia

SRBCT small round blue-cell tumor

EWS Ewing family of tumors

RMS rhabdomyosarcoma

NB neuroblastoma

NHL non-Hodgkin lymphoma

ANN artificial neural network

RJMCMC reversible jump Markov chain Monte Carlo

IG inverted gamma

PMF probability mass function

RF random forests

DLDA diagonal linear discriminant analysis

## Authors' contributions

LWKC conceived and supervised this study. XZ performed the computational work and analyzed the data. Both authors contributed to the designing and developing of the methodology and computational/statistical strategy. Both authors contributed to the writing of the manuscript.

## Appendix: Inference for *P*(t|z, *b*, K_*γ*_)

First of all, for convenience, we drop the notation of *b *and **K**_**γ **_in the following derivations. Under an KIGP model, we have

**t **~ *N *(**0**, **K**_**γ**_), **z**|**t **~ *N*(**t **+ *b***l_n_**, **Ω**), where **K**_**γ**_, **l**_**n **_and **Ω **are defined in Eq. (12).     (A1)

The joint distribution of **z **and **t **is still Gaussian, which can be formulated as:

P(z,t)=P(z|t)P(t)∝exp⁡{−12[(z−t−b1n)'Ω−1(z−t−b1n)+t'Kγ−1t)∝exp⁡{−12[(t−μt)'(Kγ−1+Ω−1)(t−μt)+(z−b1n)'Ω−1(z−b1n)−μt'(Kγ−1+Ω−1)μt]}
 MathType@MTEF@5@5@+=feaafiart1ev1aaatCvAUfKttLearuWrP9MDH5MBPbIqV92AaeXatLxBI9gBaebbnrfifHhDYfgasaacH8akY=wiFfYdH8Gipec8Eeeu0xXdbba9frFj0=OqFfea0dXdd9vqai=hGuQ8kuc9pgc9s8qqaq=dirpe0xb9q8qiLsFr0=vr0=vr0dc8meaabaqaciaacaGaaeqabaqabeGadaaakqaabeqaaiabdcfaqjabcIcaOiabhQha6jabcYcaSiabhsha0jabcMcaPiabg2da9iabdcfaqjabcIcaOiabhQha6jabcYha8jabhsha0jabcMcaPiabdcfaqjabcIcaOiabhsha0jabcMcaPiabg2Hi1kGbcwgaLjabcIha4jabcchaWjabcUha7jabgkHiTmaalaaabaGaeGymaedabaGaeGOmaidaaiabcUfaBjabcIcaOiabhQha6jabgkHiTiabhsha0jabgkHiTiabdkgaIjabhgdaXmaaBaaaleaacqWHUbGBaeqaaOGaeiykaKIaei4jaCccceGae8xQdC1aaWbaaSqabeaacqGHsislcqaIXaqmaaGccqGGOaakcqWH6bGEcqGHsislcqWH0baDcqGHsislcqWGIbGycqWHXaqmdaWgaaWcbaGaeCOBa4gabeaakiabcMcaPiabgUcaRiabhsha0jabcEcaNiabhUealnaaDaaaleaaiiGacqGFZoWzaeaacqGHsislcqaIXaqmaaGccqWH0baDcqGGPaqkaeaacqGHDisTcyGGLbqzcqGG4baEcqGGWbaCcqGG7bWEcqGHsisldaWcaaqaaiabigdaXaqaaiabikdaYaaacqGGBbWwcqGGOaakcqWH0baDcqGHsislcqWF8oqBdaWgaaWcbaGaeCiDaqhabeaakiabcMcaPiabcEcaNiabcIcaOiabhUealnaaDaaaleaacqGFZoWzaeaacqGHsislcqaIXaqmaaGccqGHRaWkcqWFPoWvdaahaaWcbeqaaiabgkHiTiabigdaXaaakiabcMcaPiabcIcaOiabhsha0jabgkHiTiab=X7aTnaaBaaaleaacqWH0baDaeqaaOGaeiykaKIaey4kaSIaeiikaGIaeCOEaONaeyOeI0IaemOyaiMaeCymaeZaaSbaaSqaaiabh6gaUbqabaGccqGGPaqkcqGGNaWjcqWFPoWvdaahaaWcbeqaaiabgkHiTiabigdaXaaakiabcIcaOiabhQha6jabgkHiTiabdkgaIjabhgdaXmaaBaaaleaacqWHUbGBaeqaaOGaeiykaKIaeyOeI0Iae8hVd02aa0baaSqaaiabhsha0bqaaiabcEcaNaaakiabcIcaOiabhUealnaaDaaaleaacqGFZoWzaeaacqGHsislcqaIXaqmaaGccqGHRaWkcqWFPoWvdaahaaWcbeqaaiabgkHiTiabigdaXaaakiabcMcaPiab=X7aTnaaBaaaleaacqWH0baDaeqaaOGaeiyxa0LaeiyFa0haaaa@C090@

where **μ**_**t **_= (**Ω**^-1 ^+ Kγ−1
 MathType@MTEF@5@5@+=feaafiart1ev1aaatCvAUfKttLearuWrP9MDH5MBPbIqV92AaeXatLxBI9gBaebbnrfifHhDYfgasaacH8akY=wiFfYdH8Gipec8Eeeu0xXdbba9frFj0=OqFfea0dXdd9vqai=hGuQ8kuc9pgc9s8qqaq=dirpe0xb9q8qiLsFr0=vr0=vr0dc8meaabaqaciaacaGaaeqabaqabeGadaaakeaaieqacqWFlbWsdaqhaaWcbaacciGae43SdCgabaGaeyOeI0IaeGymaedaaaaa@3188@)^-1^**Ω**^-1^(**z **- *b***l_n_**).     (A2)

In principle, if **z **and **t **form a joint Gaussian distribution, both the marginal distribution of **z **and the conditional distribution of **t **given **z **are also Gaussian. Making use of the following equation from [33]:

(**A **+ **C**)^-1 ^= **A**^-1 ^- **A**^-1^(**A**^-1 ^+ **C**^-1^)^-1^**A**^-1^,     (A3)

it consequently yields

P(z)=∫tP(z,t)dt∝exp⁡{−12[(z−b1n)'Ω−1(z−b1n)−μt'(Kγ−1+Ω−1)μt]}∝exp⁡{−12[(z−b1n)'(Kγ+Ω)−1(z−b1n)]}     (A4)
 MathType@MTEF@5@5@+=feaafiart1ev1aaatCvAUfKttLearuWrP9MDH5MBPbIqV92AaeXatLxBI9gBaebbnrfifHhDYfgasaacH8akY=wiFfYdH8Gipec8Eeeu0xXdbba9frFj0=OqFfea0dXdd9vqai=hGuQ8kuc9pgc9s8qqaq=dirpe0xb9q8qiLsFr0=vr0=vr0dc8meaabaqaciaacaGaaeqabaqabeGadaaakeaafaqabeGabaaabaGaemiuaaLaeiikaGIaeCOEaONaeiykaKIaeyypa0Zaa8quaeaacqWGqbaucqGGOaakcqWH6bGEcqGGSaalcqWH0baDcqGGPaqkaSqaaiabhsha0bqab0Gaey4kIipakiabdsgaKjabhsha0jabg2Hi1kGbcwgaLjabcIha4jabcchaWjabcUha7jabgkHiTmaalaaabaGaeGymaedabaGaeGOmaidaaiabcUfaBjabcIcaOiabhQha6jabgkHiTiabdkgaIjabhgdaXmaaBaaaleaacqWHUbGBaeqaaOGaeiykaKIaei4jaCccceGae8xQdC1aaWbaaSqabeaacqGHsislcqaIXaqmaaGccqGGOaakcqWH6bGEcqGHsislcqWGIbGycqWHXaqmdaWgaaWcbaGaeCOBa4gabeaakiabcMcaPiabgkHiTiab=X7aTnaaDaaaleaacqWH0baDaeaacqGGNaWjaaGccqGGOaakcqWHlbWsdaqhaaWcbaacciGae43SdCgabaGaeyOeI0IaeGymaedaaOGaey4kaSIae8xQdC1aaWbaaSqabeaacqGHsislcqaIXaqmaaGccqGGPaqkcqWF8oqBdaWgaaWcbaGaeCiDaqhabeaakiabc2faDjabc2ha9bqaaiabg2Hi1kGbcwgaLjabcIha4jabcchaWjabcUha7jabgkHiTmaalaaabaGaeGymaedabaGaeGOmaidaaiabcUfaBjabcIcaOiabhQha6jabgkHiTiabdkgaIjabhgdaXmaaBaaaleaacqWHUbGBaeqaaOGaeiykaKIaei4jaCIaeiikaGIaeC4saS0aaSbaaSqaaiab+n7aNbqabaGccqGHRaWkcqWFPoWvcqGGPaqkdaahaaWcbeqaaiabgkHiTiabigdaXaaakiabcIcaOiabhQha6jabgkHiTiabdkgaIjabhgdaXmaaBaaaleaacqWHUbGBaeqaaOGaeiykaKIaeiyxa0LaeiyFa0haaiaaxMaacaWLjaWaaeWaaeaacqqGbbqqcqaI0aanaiaawIcacaGLPaaaaaa@A246@

and

P(t|z)∝exp⁡{−12[(t−μt)'(Kγ−1+Ω−1)(t−μt)]}p∝exp⁡{−12[(t−μt)'(Ω−Ω(Ω+Kγ)−1Ω)−1(t−μt)]}
 MathType@MTEF@5@5@+=feaafiart1ev1aaatCvAUfKttLearuWrP9MDH5MBPbIqV92AaeXatLxBI9gBaebbnrfifHhDYfgasaacH8akY=wiFfYdH8Gipec8Eeeu0xXdbba9frFj0=OqFfea0dXdd9vqai=hGuQ8kuc9pgc9s8qqaq=dirpe0xb9q8qiLsFr0=vr0=vr0dc8meaabaqaciaacaGaaeqabaqabeGadaaakeGadea7baaRcaaKjuaabaqaceaaaeaacqWGqbaucqGGOaakcqWH0baDcqGG8baFcqWH6bGEcqGGPaqkcqGHDisTcyGGLbqzcqGG4baEcqGGWbaCcqGG7bWEcqGHsisldaWcaaqaaiabigdaXaqaaiabikdaYaaacqGGBbWwcqGGOaakcqWH0baDcqGHsisliiqacqWF8oqBdaWgaaWcbaGaeCiDaqhabeaakiabcMcaPiabcEcaNiabcIcaOiabhUealnaaDaaaleaaiiGacqGFZoWzaeaacqGHsislcqaIXaqmaaGccqGHRaWkcqWFPoWvdaahaaWcbeqaaiabgkHiTiabigdaXaaakiabcMcaPiabcIcaOiabhsha0jabgkHiTiab=X7aTnaaBaaaleaacqWH0baDaeqaaOGaeiykaKIaeiyxa0LaeiyFa0NaemiCaahabaGaaCzcaiabg2Hi1kGbcwgaLjabcIha4jabcchaWjabcUha7jabgkHiTmaalaaabaGaeGymaedabaGaeGOmaidaaiabcUfaBjabcIcaOiabhsha0jabgkHiTiab=X7aTnaaBaaaleaacqWH0baDaeqaaOGaeiykaKIaei4jaCIaeiikaGIae8xQdCLaeyOeI0Iae8xQdCLaeiikaGIae8xQdCLaey4kaSIaeC4saS0aaSbaaSqaaiab+n7aNbqabaGccqGGPaqkdaahaaWcbeqaaiabgkHiTiabigdaXaaakiab=L6axjabcMcaPmaaCaaaleqabaGaeyOeI0IaeGymaedaaOGaeiikaGIaeCiDaqNaeyOeI0Iae8hVd02aaSbaaSqaaiabhsha0bqabaGccqGGPaqkcqGGDbqxcqGG9bqFaaaaaa@9174@

where **μ**_**t **_= (**I**_**n **_- **Ω**(**Ω **+ **K**_**γ**_)^-1^(**z **- *b***l_n_**).     (A5)

**z **~ *N*(*b***l_n_**, **K**_*γ *_+ **Ω**)

Or strictly,

**t**|**z **~ *N*((**I**_**n **_- **Ω**(**Ω **+ **K**_**γ**_)^-1^)(**z **- *b***l_n_**), **Ω **- **Ω**(**Ω **+ **K**_*γ*_)^-1^**Ω**)     (A6)

NOTE: The matrix **Ω **- **Ω**(**Ω **+ **K**_*γ*_)^-1^**Ω **is non-negative definite.
